# C3G contributes to platelet activation and aggregation by regulating major signaling pathways

**DOI:** 10.1038/s41392-020-0119-9

**Published:** 2020-04-01

**Authors:** Sara Gutiérrez-Herrero, Cristina Fernández-Infante, Luis Hernández-Cano, Sara Ortiz-Rivero, Carlos Guijas, Víctor Martín-Granado, José Ramón González-Porras, Jesús Balsinde, Almudena Porras, Carmen Guerrero

**Affiliations:** 1 Instituto de Biología Molecular y Celular del Cáncer (IMBCC), University of Salamanca-CSIC, Salamanca, Spain; 2 Instituto de Investigación Biomédica de Salamanca (IBSAL), Salamanca, Spain; 3 Instituto de Biología y Genética Molecular (IBGM), Consejo Superior de Investigaciones Científicas (CSIC), University of Valladolid, Valladolid, Spain; 4 Centro de Investigación Biomédica en Red de Diabetes y Enfermedades Metabólicas Asociadas (CIBERDEM), Madrid, Spain; 5 Departamento de Hematología, Hospital Universitario de Salamanca (HUS), Salamanca, Spain; 6 Departamento de Bioquímica y Biología Molecular, Facultad de Farmacia, Complutense University of Madrid. Instituto de Investigación Sanitaria del Hospital Clínico San Carlos (IdISSC), Madrid, Spain; 7 Departamento de Medicina, University of Salamanca, Salamanca, Spain

**Keywords:** Cell biology, Molecular medicine

## Abstract

C3G is a GEF (guanine nucleotide exchange factor) for Rap GTPases, among which the isoform Rap1b is an essential protein in platelet biology. Using transgenic mouse models with platelet-specific overexpression of C3G or mutant C3GΔCat, we have unveiled a new function of C3G in regulating the hemostatic function of platelets through its participation in the thrombin-PKC-Rap1b pathway. C3G also plays important roles in angiogenesis, tumor growth, and metastasis through its regulation of the platelet secretome. In addition, C3G contributes to megakaryopoiesis and thrombopoiesis. Here, we used a platelet-specific C3G-KO mouse model to further support the role of C3G in hemostasis. C3G-KO platelets showed a significant delay in platelet activation and aggregation as a consequence of the defective activation of Rap1, which resulted in decreased thrombus formation in vivo. Additionally, we explored the contribution of C3G-Rap1b to platelet signaling pathways triggered by thrombin, PMA or ADP, in the referenced transgenic mouse model, through the use of a battery of specific inhibitors. We found that platelet C3G is phosphorylated at Tyr504 by a mechanism involving PKC-Src. This phosphorylation was shown to be positively regulated by ERKs through their inhibition of the tyrosine phosphatase Shp2. Moreover, C3G participates in the ADP-P2Y12-PI3K-Rap1b pathway and is a mediator of thrombin-TXA_2_ activities. However, it inhibits the synthesis of TXA_2_ through cPLA_2_ regulation. Taken together, our data reveal the critical role of C3G in the main pathways leading to platelet activation and aggregation through the regulation of Rap1b.

## Introduction

C3G, also known as RapGEF1, is the primary activator of Rap1 GTPases, among which the Rap1b isoform plays a critical role in most platelet functions.^1^ Using transgenic mouse models specifically expressing full-length human C3G or mutant human C3G∆Cat (lacking the catalytic domain) in megakaryocytes (MKs) and platelets, we have unveiled an important function of C3G in platelet biology. Transgenic expression of C3G in platelets stimulates (i) megakaryocytic differentiation and proplatelet formation,^2^ (ii) platelet activation and aggregation,^3^ and (iii) α-granule secretion and platelet angiogenesis and metastasis.^4^ In addition, we have shown that C3G participates in the activation of Rap1 triggered by thrombin and PMA (phorbol 12-myristate 13-acetate) through the PKC pathway.^3^ Other investigations have demonstrated the formation of a ternary VASP/CrkL/C3G complex that activates platelet Rap1b.^5^

Most platelet agonists, such as thrombin, ADP (adenosine diphosphate), TXA_2_ (thromboxane A2) or epinephrine, stimulate GPCR-type surface receptors, while others, such as collagen and vWF (von Willebrand factor), stimulate immunoglobulin-like receptors. These various receptors trigger signaling cascades that converge in the activation of Rap1b, the most abundant GTPase in platelets.^6,7^ Thus, through its interaction with PAR-1 and PAR-4, thrombin activates Rap1b via two sequential activation waves involving PLC-CalDAG-GEFI and PKC-C3G.^3,8^ On the other hand, ADP, through its binding to purinergic P2Y12 receptors coupled with Gαi, stimulates Rap1b activation via a calcium-independent pathway that engages PI3K,^9–11^ although the GEF for Rap1b that mediates PI3K activities in platelets remains to be identified.^7^

TXA_2_ also participates in the activation of Rap1 through its release and subsequent binding to TXA_2_ receptors. It has become increasingly clear that MAPKs participate in the synthesis of TXA_2_ by activating cPLA_2_.^12^ However, the precise contribution of MAPKs to other platelet functions remains controversial. Some studies suggest that both ERKs and p38 MAPKs are essential for platelet spreading, aggregation, thrombus formation and α-granule secretion,^6,13^ while others have suggested that these kinases do not contribute to platelet activation or aggregation.^14^ Neither of the two Rap1 isoforms mediates ERK or p38 MAPK activation in platelets,^1,15^ although a negative role for Rap1 in ERK and p38 MAPK activation has been demonstrated in various cell types, including hematopoietic cells.^16^ Moreover, in platelets, Raf-1 and B-Raf do not participate in the activation of ERK2 by thrombin, a role that has been attributed to PKC.^17^

Full C3G activation requires its phosphorylation at residue Tyr504 by SFKs (Src-family kinases),^18^ which participate in the responses of most platelet agonists. In platelets, Src and PKC are reciprocally activated by transphosphorylation. Specifically, PKC phosphorylates Src at Ser12, a residue involved in cytoskeletal association and substrate affinity.^19^

Src does not contribute to the activation of ERKs induced by thrombin.^17^ However, ERKs are downstream targets of SFKs in the signaling pathway initiated by the binding of vWF to the GPIb-IX-V receptor, leading to TXA_2_ generation.^19^ Moreover, in MKs under thrombopoietin (TPO) stimulation, the SFK Lyn reduced ERK activity through the activation of PTPs (protein tyrosine phosphatases) such as Shp1.^20^ In fact, the Shp1 and Shp2 phosphatases are major regulators of megakaryopoiesis, thrombopoiesis, and platelet function.^21^ Recent studies showed an increase in the phosphorylation levels of ERKs in Shp2^−/−^ platelets,^21,22^ suggesting the negative role of Shp2 in ERK activation. However, in other hematopoietic cells, Shp2 acts as a positive regulator of the Ras-ERK pathway downstream of cytokines and RTK receptors.^23^ In addition, ERKs were found to phosphorylate and inhibit Shp2 in pheochromocytoma PC12 cells in vitro.^24^

In this work, we confirmed the role of C3G in Rap1-mediated platelet activation and aggregation using a conditional knockout model. In addition, through the use of specific inhibitors, we explored the participation of transgenic (tg) C3G in platelet signaling pathways triggered by thrombin, PMA and ADP by three different approaches: (i) study of the differential sensitivities of tgC3G and tgC3GΔCat platelets to the inhibition of platelet activation and aggregation, (ii) study of the phosphorylation of C3G at Tyr504 by immunofluorescence confocal microscopy, and (iii) analysis of the activation of Rap1b in platelets of the different genotypes used. Our results support the participation of C3G in signaling pathways regulated by PKC-Src, PI3K, MAPKs and TXA_2_, as well as its role in the modulation of TXA_2_ synthesis through a negative feedback mechanism involving cPLA_2_.

## Results

### Platelets lacking C3G show major defects in platelet activation and aggregation

Successful abrogation of C3G in MKs and platelets was verified by analyses of genomic DNA in MKs and mRNA and protein expression in MKs and platelets (Fig. S1a–d). Some residual C3G mRNA was detected in KO samples, which probably came from contaminating cells.

**Fig. 1:**
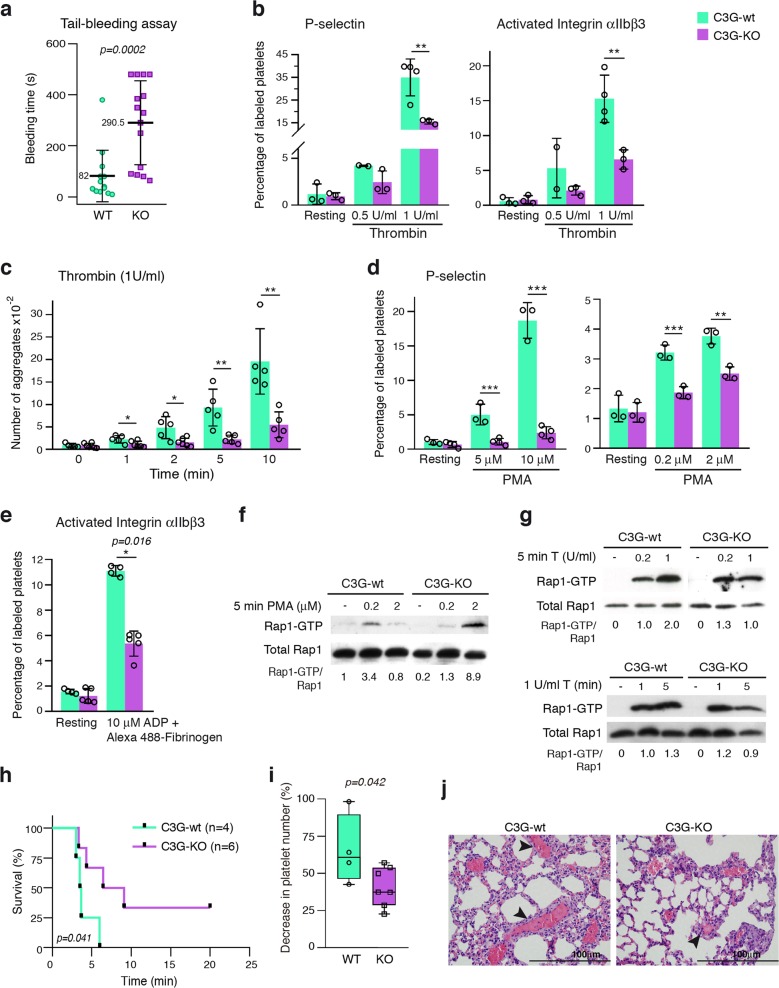
The targeting of C3G in platelets decreases Rap1 activation, resulting in impaired platelet activation and aggregation. **a** Tail-bleeding assays were performed in C3G-KO (KO) mice and their wild-type (WT) controls at weaning. The scatter plots represent the bleeding times of 13–15 animals of each genotype. The mean values are shown. The Mann–Whitney *U* test was performed. **b** Washed blood from C3G-KO and C3G-wt mice was stimulated with 0.5 U/ml or 1 U/ml thrombin and incubated with anti-CD62P-FITC to determine the percentage of platelets presenting P-selectin on their surface or with JON/A-PE antibody to determine the percentage of platelets in which the integrin αIIbβ3 was activated. The histograms represent the mean ± SD of the percentage of labeled platelets. **c** Histograms represent the mean ± SD of the number of platelet aggregates formed upon 1 U/ml thrombin stimulation for the indicated time periods. **d** Washed blood from C3G-KO and C3G-wt mice was stimulated with PMA at the indicated concentrations and incubated with anti-CD62P-FITC. The histograms represent the mean ± SD of the percentage of labeled platelets. **e** Washed blood from C3G-KO and C3G-wt mice was stimulated with 10 μM ADP in combination with Alexa 488-fibrinogen, and labeled platelets were detected by flow cytometry. The histograms represent the mean ± SD of the percentage of labeled platelets. Platelets from C3G-KO and C3G-wt mice were stimulated with (**f**) 0.2 or 2 μM PMA for 5 min, (**g**) 0.2 or 1 U thrombin for 5 min (upper) or 1 U thrombin for 1 or 5 min (lower). The levels of Rap1-GTP were determined by pulldown assay and immunoblotting. Values are relative to the control value (**f**) or to the value for thrombin in wild-type platelets (**g**) and were normalized against total Rap1. The Rap1-GTP/Total Rap1 ratio is indicated beneath the blots. **h** Kaplan–Meier survival plots for mice after the induction of acute pulmonary thromboembolism (i.v. injection of a lethal dose of collagen/norepinephrine). Survival was significantly increased in C3G-KO mice (*p* = 0.0409, log-rank test). Two of six C3G-KO mice survived the lethal treatment. **i** The histograms show the percentage of reduction in blood platelets induced by the i.v. injection of a sublethal dose of collagen/norepinephrine. Blood was withdrawn before the injection and 4 min after injection. The Mann–Whitney *U* test was performed. **j** Representative H&E staining of lung sections from mice treated with a lethal dose of collagen/norepinephrine. Thrombi (arrowheads) appeared as eosinophilic material with borders, with partial or total affectation of the lumen of the small and medium caliber blood vessels located mainly on the periphery of the pulmonary lobes. Bar: 100 μm. *p < 0.05, **p < 0.01, ***p < 0.001. wt: wild-type; T: thrombin.

Tail-bleeding assays performed in *Rapgef1*^flox/flox^;PF4-Cre^+/−^ (hereinafter C3G-KO) mice and their controls (*Rapgef1*^flox/flox^;PF4-Cre^−/−^ (hereinafter C3G-wt) mice) revealed a significant bleeding diathesis in C3G-KO mice that was not observed in C3G-wt siblings (Fig. 1a), a phenotype similar to that of transgenic C3GΔCat mice.^3^ In addition, C3G-KO platelets showed impaired platelet activation and aggregation in response to thrombin, PMA and ADP (Fig. 1b–e and Fig. S1e). This occurred in conjunction with defective Rap1 activation in response to either PMA (Fig. 1f) or high-dose thrombin (1 U/ml) for a period of 5 min (Fig. 1g). These results suggest that C3G is the main Rap1 activator under these conditions. This finding is in agreement with the phenotype of CalDAG-GEFI-KO platelets, which showed no significant alterations in aggregation or Rap1 activation in response to PMA or thrombin at a high dose or under long-term stimulation.^25^ The increased levels of Rap1-GTP in C3G-KO platelets upon stimulation with PMA at a high concentration reflect a delay in the activation of Rap1 and hence its slower downregulation.^8^ To further demonstrate in vivo the impact of C3G ablation in platelets, we developed a model of pulmonary thromboembolism by injecting a mixture of collagen/epinephrine into the jugular vein of C3G-KO and C3G-wt mice. C3G-KO mice given collagen/epinephrine at a dose that induces acute pulmonary thromboembolism showed a significant increase in survival, with some animals having survived after 20 min (Fig. 1h). Injection of a sublethal dose of collagen/epinephrine produced a significantly smaller decline in the number of circulating platelets in C3G-KO mice than in C3G-wt mice (Fig. 1i), which suggests decreased aggregation. In conjunction with these results, the lungs of C3G-wt mice showed larger thrombi than those of C3G-KO animals (Fig. 1j).

### PKC indirectly activates C3G through protein tyrosine kinases

**Fig. 2:**
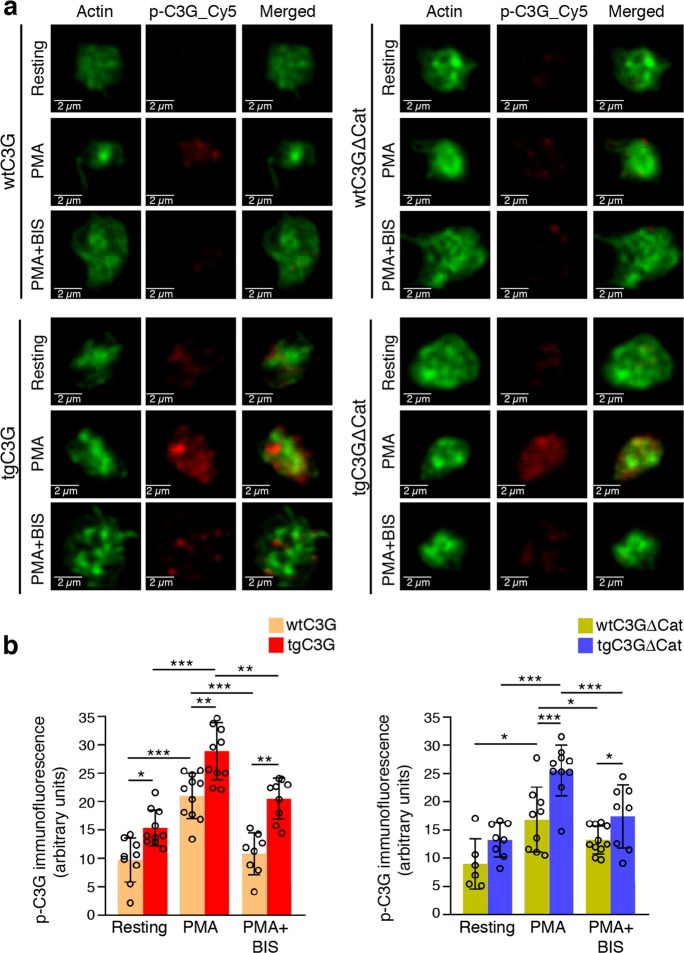
PKC promotes the phosphorylation of platelet C3G at Tyr504. tgC3G platelets, tgC3GΔCat platelets and their controls (wtC3G and wtC3GΔCat, respectively) were treated with PMA (2 μM) in the presence or absence of BIS (5 μM) and labeled with anti-pTyr504-C3G_Cy5 (red) and phalloidin (actin, green). **a** Representative immunofluorescence confocal microscopy images of platelets of each genotype under each treatment condition taken at the same exposure time. Bar: 2 μm. **b** The histograms represent the mean ± SD of the fluorescence intensity (arbitrary units) of pTyr504-C3G (p-C3G), as quantified by ImageJ. *p < 0.05; **p < 0.01; ***p < 0.001. tg: transgenic; wt: wild-type; BIS: bisindolylmaleimide.

Previously, we found that thrombin activates Rap1 in mouse platelets through the PKC-C3G pathway.^3^ The above results, which showed a substantial impairment in the PMA-PKC pathway in C3G-KO platelets, confirm these findings. Specifically, this pathway participates in the second wave of Rap1 activation induced by thrombin.^8^ Phosphorylation of C3G at Tyr504 is important for its full activation.^26^ Therefore, we determined whether PMA could induce C3G phosphorylation and whether PKC participates in this phosphorylation. For this purpose, we used confocal fluorescence techniques, which allow greater sensitivity than western blotting. As shown in Fig. 2, basal levels of pTyr504-C3G were higher in the tgC3G and tgC3GΔCat platelets than in their controls, according to the increased expression of C3G in both transgenic models. pTyr504-C3G levels were further increased upon PMA stimulation, which was almost fully inhibited by the PKC inhibitor BIS (bisindolylmaleimide), indicating that the phosphorylation of C3G in response to this stimulus was dependent on PKC. The specificity of the pTyr504-C3G signal was validated by its colocalization with the total C3G signal (Fig. S2).

This result indicates that PKC stimulates C3G activation in platelets. However, since PKC is a Ser/Thr kinase, its effect on the phosphorylation of C3G at Tyr504 must be indirect.

### Src phosphorylates C3G at Tyr504 in thrombin-stimulated platelets

**Fig. 3:**
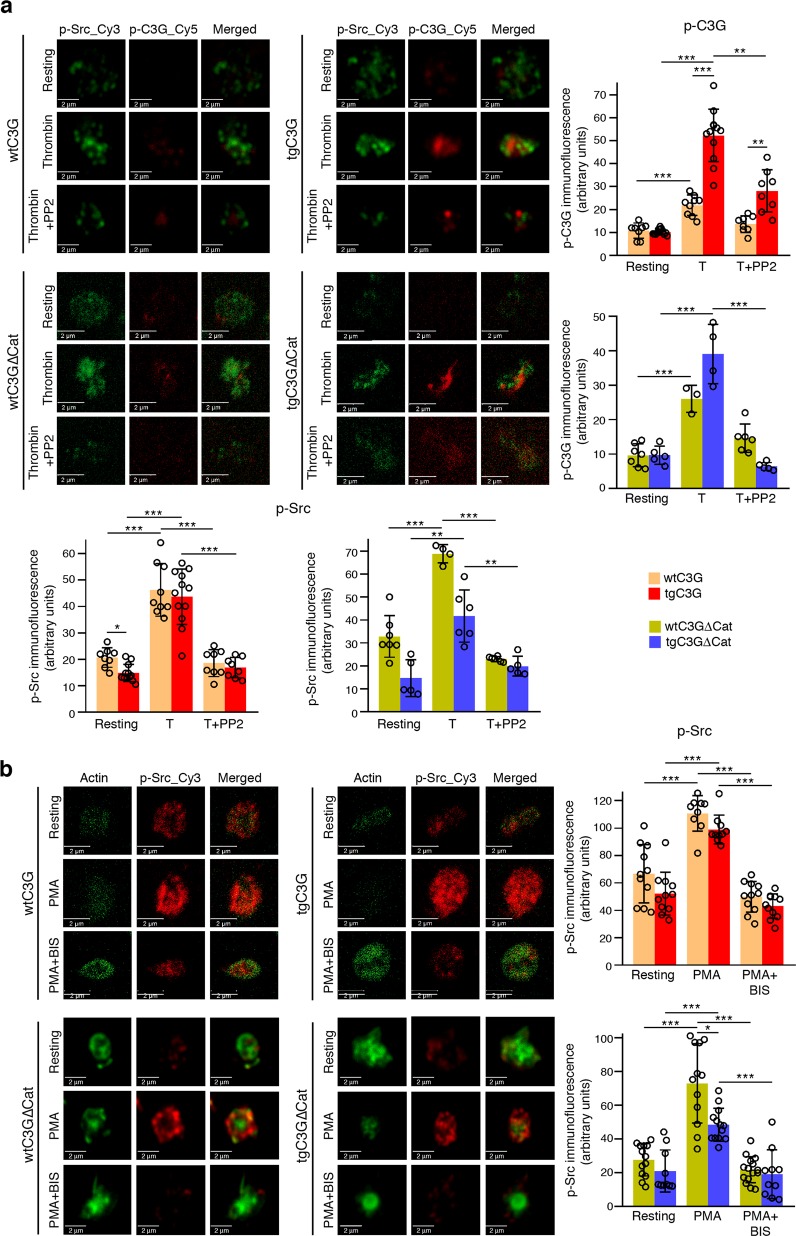
The thrombin-PKC pathway engages Src to phosphorylate C3G at Tyr504. **a** tgC3G platelets, tgC3GΔCat platelets and their controls (wtC3G and wtC3GΔCat, respectively) were treated with thrombin (0.5 U/ml) in the presence or absence of PP2 (10 μM) and labeled with anti-pTyr418-Src_Cy3 (green) and anti-pTyr504-C3G_Cy5 (red). Upper left: representative immunofluorescence confocal microscopy images of platelets of each genotype under each treatment condition taken at the same exposure time. Bar: 2 μm. Histograms represent the mean ± SD of the fluorescence intensities (arbitrary units) of pTyr504-C3G (p-C3G, upper right panels) and pTyr418-Src (p-Src, lower panels), as quantified by ImageJ. T: thrombin. **b** tgC3G platelets, tgC3GΔCat platelets and their controls were stimulated with PMA (2 μM) in the presence or absence of BIS (5 μM) and labeled with anti-pTyr418-Src_Cy5 (red) and phalloidin (green). Left: representative immunofluorescence confocal microscopy images of platelets of each genotype under each treatment condition taken at the same exposure time. Bar: 2 μm. Right: histograms represent the mean ± SD of the fluorescence intensity (arbitrary units) of p-Src relative to the phalloidin signal, as quantified by ImageJ. *p < 0.05, **p < 0.01, ***p < 0.001. tg: transgenic; wt: wild-type; BIS: bisindolylmaleimide.

SFKs, such as Src, Fyn and Hck, and other nonreceptor tyrosine kinases, such as Abl, are capable of phosphorylating C3G at Tyr504 in response to different stimuli.^18,27–29^ Therefore, we studied whether Src is the kinase responsible for the phosphorylation of C3G induced by the thrombin-PKC pathway in platelets. As shown in Fig. 3a, thrombin clearly stimulated Tyr504-C3G phosphorylation, mainly in the transgenic platelets, which was almost completely abrogated by the specific SFK inhibitor PP2. In addition, both thrombin and PMA induced the phosphorylation of Src at Tyr418 (Tyr424 in mice), a residue responsible for its activation (Fig. 3a, b). Moreover, PMA-induced Tyr418-Src phosphorylation was inhibited by pretreatment with BIS (Fig. 3b). All these results suggest that Src is the kinase that phosphorylates C3G during the second wave of thrombin stimulation involving PKC. The phosphorylation of Src appears to be independent of the overexpression of C3G and C3GΔCat, based on the location of Src upstream of the C3G-Rap1 pathway.

### C3G participates in platelet pathways triggered by ADP

We previously reported that tgC3G platelets showed increased activation and aggregation after stimulation with ADP.^3^ ADP is released from dense granules in response to thrombin to amplify platelet responses through interacting with its specific receptors P2Y1 and P2Y12.^30^ To gain in-depth knowledge into the participation of C3G in platelet pathways initiated by ADP, we analyzed the activation and aggregation of platelets from tgC3G, tgC3GΔCat and control mice after the inhibition of these ADP receptors with clopidogrel (a P2Y12 inhibitor) or MRS2179 (a P2Y1 inhibitor), followed by thrombin or ADP stimulation. Previously, we verified the inhibitory effects of these inhibitors and others used in this work on thrombin-induced platelet activation and aggregation (Fig. S3).

**Fig. 4:**
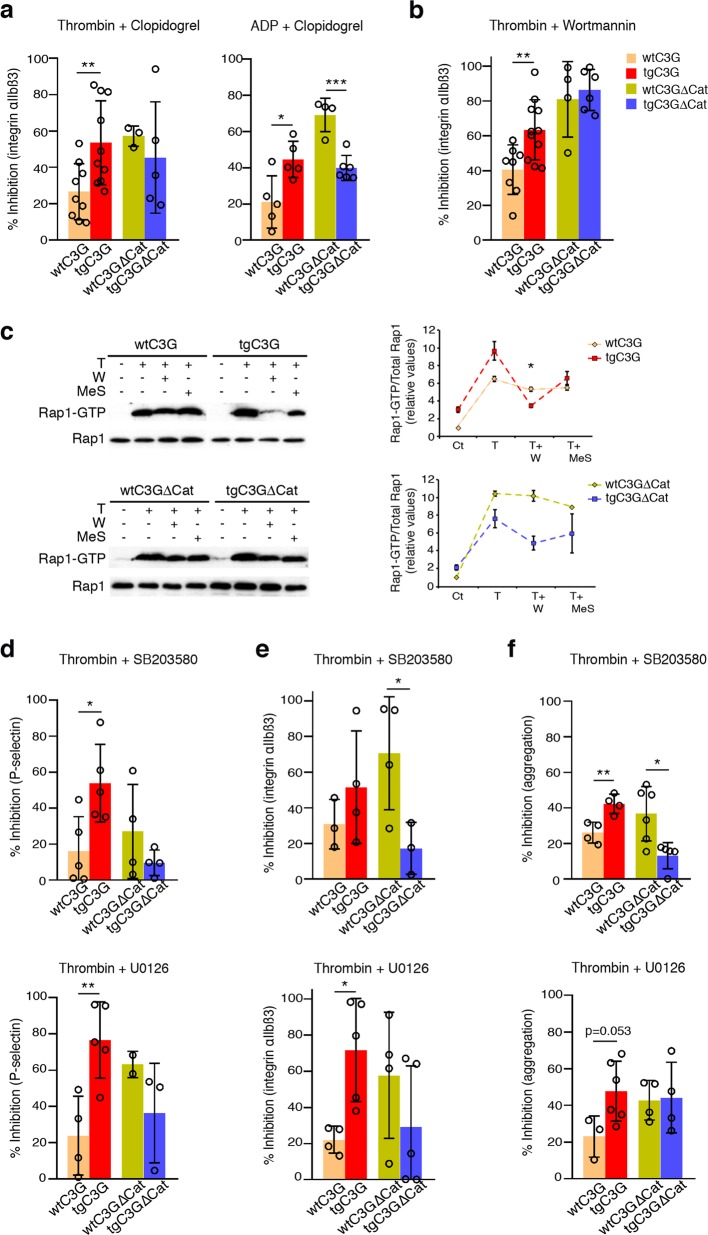
TgC3G and tgC3GΔCat platelets show differential sensitivities to inhibition of the P2Y12, PI3K, ERK and p38 MAPK signaling pathways. **a** Platelets from mice of the different genotypes under study were pretreated with 100 μM clopidogrel for 5 min and then stimulated for 15 min with 1 U/ml thrombin or 10 μM ADP, as indicated. The histograms represent the mean ± SD of the percentage of inhibition of activated integrin αIIbβ3 in platelets treated with agonist + inhibitor compared to platelets treated with agonist. **b** Platelets were pretreated with 100 nM wortmannin for 5 min and then stimulated with 1 U/ml thrombin for 15 min. The histograms represent the mean ± SD of the percentage of inhibition of activated integrin αIIbβ3 in platelets treated with agonist + inhibitor compared to platelets treated with agonist. **c** Platelets were pretreated with 100 nM wortmannin or 50 μM 2-MeSAMP for 5 min and then stimulated with 0.5 U/ml thrombin for 5 min. Rap1-GTP was isolated by pulldown with GST-RalGDS-RBD and detected by immunoblotting with anti-Rap1 antibodies. Left panel: representative western blots. Right panel: line/scatter plots of Rap1-GTP levels (*n* = 2). Values (mean ± SEM) are relative to those in unstimulated wild-type platelets and were normalized to total Rap1 levels. **d**–**f** Platelets were pretreated with 20 μM U0126 or SB203580 for 5 min and then stimulated with 1 U/ml thrombin (15 min for activation, 10 min for aggregation), as indicated. The histograms represent the mean ± SD of the percentages of inhibition of the expression of P-selectin on the surface (**d**), activation of integrin αIIbβ3 (**e**) or aggregation (**f**) in platelets treated with thrombin + inhibitor, compared to thrombin-treated platelets. *p < 0.05, **p < 0.01, ***p < 0.001. tg: transgenic; wt: wild-type; T: thrombin; W: wortmannin; MeS: 2-MeSAMP.

For this study, and since C3G-KO platelets are barely activated, we monitored the sensitivity of tgC3G and tgC3GΔCat platelets and their controls to the inhibitory effect of clopidogrel and MRS2179. Our hypothesis was that the transgenic expression of C3G would make platelets more dependent on C3G. Therefore, they would be more sensitive to inhibition of the pathways in which C3G participates, leading to increased inhibition. Indeed, the inhibitory effect of clopidogrel on the activation of integrin αIIbβ3 after stimulation with thrombin or ADP was significantly greater in tgC3G platelets than in wtC3G platelets (Fig. 4a). A similar tendency was observed for the inhibition of P-selectin expression on the surface and the aggregation induced by thrombin (Fig. S4a). In contrast, tgC3GΔCat platelets tended to be more resistant to the activities of these inhibitors than the corresponding control platelets.

Inhibition of the ADP receptor P2Y1 also differentially affected tgC3G platelets compared to wtC3G platelets, although to a lesser extent than inhibition of the P2Y12 receptor, since the differences were not significant. Similarly, there was no differential effect on tgC3GΔCat platelets compared to their controls (Fig. S4b). This suggests a minor contribution of C3G to this pathway. All these results indicate that C3G participates in platelet pathways activated by ADP, mainly through the P2Y12 receptor.

ADP engages P2Y12 receptors to activate Rap1 through a pathway involving PI3K.^10^ In fact, similar to what was observed with clopidogrel, tgC3G platelets were significantly more sensitive than control platelets to the inhibitory effect of wortmannin (a PI3K inhibitor) on thrombin-induced integrin αIIbβ3 activation (Fig. 4b).

Next, we analyzed the levels of Rap1-GTP in the lysates of platelets pretreated with 2-MeSAMP (a P2Y12 receptor inhibitor) or wortmannin prior to thrombin stimulation. As shown in Fig. 4c, thrombin increased Rap1-GTP levels to a greater extent in tgC3G platelets than in wtC3G platelets, consistent with our previous results.^3^ However, tgC3G platelets were more sensitive to the activities of these inhibitors, mainly wortmannin, than wild-type or tgC3GΔCat platelets. Notably, tgC3G platelets showed less total Rap1 than their control platelets from an equivalent amount of blood (see also Figs. 6c and 7f). This is not an effect of C3G on Rap1 expression, since correlative variation between actin levels was observed (data not shown). It is plausible that the increased aggregation of these platelets decreases their availability; the opposite scenario applies to tgC3GΔCat platelets.

Overall, these results suggest that C3G participates in the ADP-P2Y12-PI3K-Rap1 pathway.

### C3G contributes to MAPK-mediated activities, leading to platelet activation and aggregation

Several studies have suggested the participation of ERKs and p38 MAPKs in most platelet functions, including platelet aggregation and thrombus formation,^31,32^ α-granule secretion^33^, and TXA_2_ synthesis.^12^ In platelets, ERK activation is independent of Raf-1 and B-Raf but dependent on PKC and Src.^12,17,34^ Based on the functional relationships between C3G and these MAPKs in other systems,^35–39^ we evaluated whether C3G and these MAPKs contribute to platelet aggregation and activation through a common pathway.

Following the same reasoning described above, we monitored the sensitivities of thrombin-stimulated tgC3G, tgC3GΔCat and control platelets to selective inhibitors of these MAPKs: U0126 (an inhibitor of MEK1/2) and SB203580 (an inhibitor of p38α/β MAPK). TgC3G platelets were more sensitive to the inhibition of ERKs and p38α/β MAPKs than wtC3G platelets, since both inhibitors (mainly U0126) produced a significantly greater reduction in the levels of P-selectin on the surface (Fig. 4d) and in the activation of integrin αIIbβ3 (Fig. 4e) in tgC3G platelets. Both inhibitors also produced an almost significant greater inhibition on tgC3G platelet aggregation, compared to wtC3G platelets (Fig. 4f). Again, tgC3GΔCat platelets tended to be more resistant to the inhibition of these MAPK pathways than control platelets.

### Transgenic C3G expression does not affect ERK and p38 MAPK activation

The above results suggest that C3G participates in ERK and p38 MAPK signaling pathways, as either an activator or a substrate. To evaluate whether C3G contributes to the activation of these MAPK pathways, we analyzed their phosphorylation status in platelets from mice of the different genotypes under study treated with thrombin for 1 min in the presence of inhibitors of several platelet signaling pathways (Fig. S5). There were no significant differences in the levels of ERK and p38 MAPK phosphorylation between the platelets from mice of the different genotypes stimulated for a short time with thrombin, in concordance with the finding that Rap1 activity does not affect the transient activation of these MAPKs.^1^ In addition, an analysis of ERK phosphorylation in K562 clones with the overexpression or knockout of C3G revealed that C3G participates in the sustained activation of ERKs, leading to K562 differentiation (Fig. S6a),^2^ but does not contribute to short-term ERK activation (Fig. S6b).

In addition, only BIS clearly inhibited thrombin-induced ERK phosphorylation, in agreement with the known role of platelet PKC in the activation of the ERK pathway (Fig. S5).^17,34^

### C3G phosphorylation is positively regulated by ERKs and p38 MAPKs

**Fig. 5:**
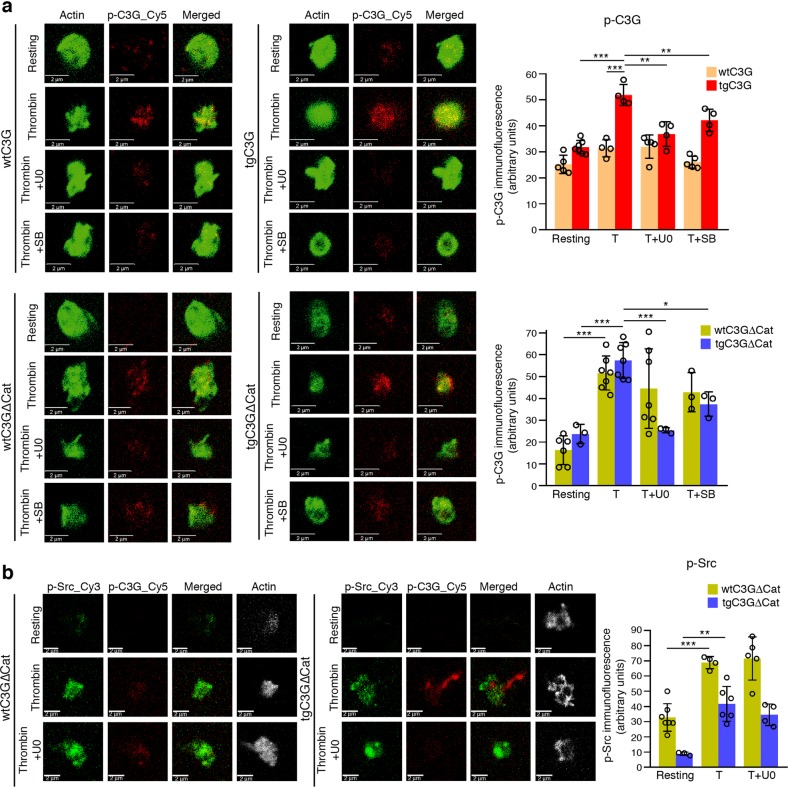
ERKs and p38 MAPKs favor the phosphorylation of C3G at Tyr504. **a** Platelets from mice of the different genotypes under study were stimulated for 1 min with thrombin (0.5 U/ml) after 5 min of treatment with U0126 (20 μM) or SB203580 (20 μM) and labeled with anti-pTyr504-C3G_Cy5 (red) and phalloidin (green). Left: representative immunofluorescence confocal microscopy images taken at the same exposure time. Bar: 2 μm. Right: histograms represent the mean ± SD of the fluorescence intensity (arbitrary units) of pTyr504-C3G (p-C3G) relative to the phalloidin signal, as quantified by ImageJ. **b** tgC3GΔCat platelets and their controls (wtC3GΔCat) were treated as described above in the presence or absence of U0126 and labeled with anti-pTyr504-C3G_Cy5 (red), pTyr418-Src_Cy3 (green) and phalloidin (white). Left: representative immunofluorescence confocal microscopy images taken at the same exposure time. Bar: 2 μm. Right: histograms represent the mean ± SD of the fluorescence intensity (arbitrary units) of pTyr418-Src (p-Src) relative to the phalloidin signal, as quantified by ImageJ. *p < 0.05, **p < 0.01, ***p < 0.001. tg: transgenic; wt: wild-type; T: thrombin; U0: U0126; SB: SB203580.

Since C3G does not seem to contribute substantially to the transient activation of ERKs and p38 MAPKs in platelets, we evaluated the possibility that C3G is downstream of these MAPKs. To that end, we studied whether ERKs and/or p38 MAPKs are involved in the regulation of C3G phosphorylation at Tyr504. Platelets were pretreated with U0126 or SB203580 prior to stimulation with thrombin, and the levels of pTyr504-C3G were analyzed by fluorescence confocal microscopy. As shown in Fig. 5a, thrombin-induced Tyr504-C3G phosphorylation was completely abolished by both inhibitors, mainly in the transgenic platelets, which suggests a positive role of ERKs and p38 MAPKs in the activation of C3G in platelets.

However, like PKC, MAPKs are Ser/Thr kinases; therefore, they cannot be directly responsible for Tyr504-C3G phosphorylation. Moreover, U0126 did not prevent Src phosphorylation at Tyr418 (Fig. 5b), indicating that Src and ERKs regulate C3G through different mechanisms.

### ERKs enhance C3G phosphorylation through inhibition of the phosphatase Sph2

**Fig. 6:**
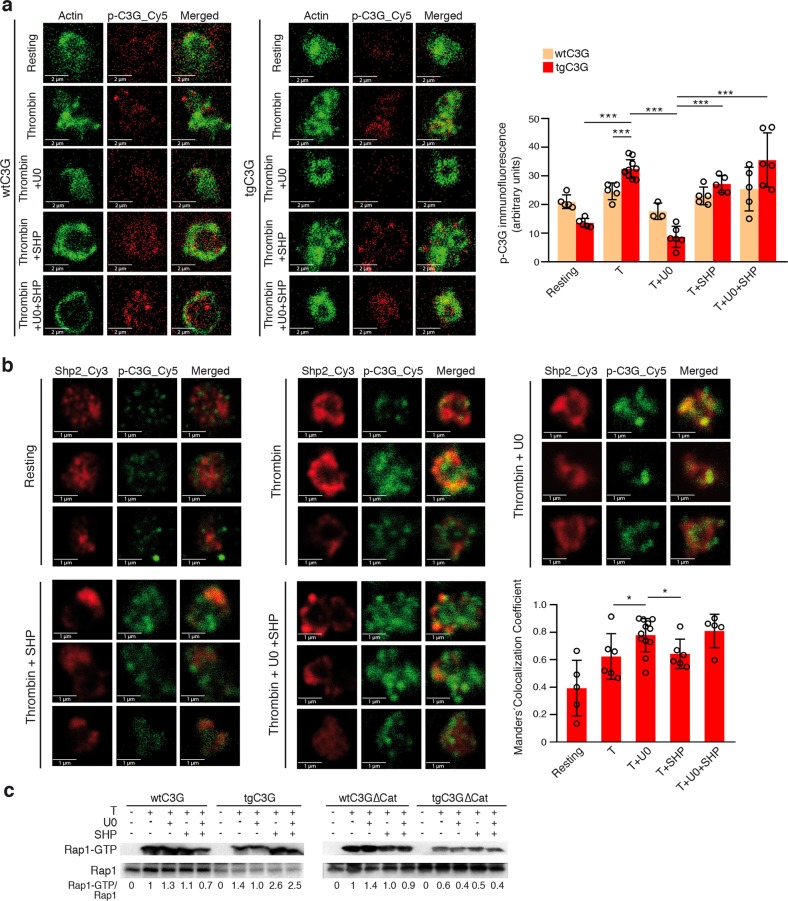
ERKs regulate the activation of C3G by acting through the phosphatase Shp2. **a** Representative immunofluorescence confocal microscopy images of tgC3G platelets and their controls under the indicated treatments [rest, thrombin (0.5 U/ml) treatment, thrombin + U0126 (20 μM) treatment, thrombin + SHP099 (20 μM) treatment and thrombin + U0126 + SHP099 treatment] labeled with anti-pTyr504-C3G_Cy5 (red) and phalloidin (green) were taken at the same exposure time. Bar: 2 μm. The histograms represent the mean ± SD of the fluorescence intensity (arbitrary units) of pTyr504-C3G relative to the phalloidin signal, as quantified by ImageJ. **b** Double immunofluorescence confocal microscopy images showing the subcellular distributions of Shp2 (red) and pTyr504-C3G (green) and an overlay of three representative tgC3G platelets under each stimulation condition [rest, thrombin (0.5 U/ml) treatment, thrombin + U0126 (20 μM) treatment, thrombin + SHP099 (20 μM) treatment and thrombin + U0126 + SHP099 treatment]. All micrographs were taken at the same exposure time. Scale bars: 1 μm. The graph shows the Manders’ colocalization coefficients (mean ± SD) of Shp2 and pTyr504-C3G under the indicated experimental conditions. (**c**) Platelets from tgC3G and tgC3GΔCat mice and their wild-type controls were pretreated for 5 min at 37 °C with 20 μM U0126 and/or SHP099 and then stimulated with 1 U/ml thrombin for 1 min. Rap1-GTP was isolated by pulldown with GST-RalGDS-RBD and detected by immunoblotting with anti-Rap1 antibodies. Values are relative to the value for thrombin in wild-type platelets and were normalized against total Rap1. The Rap1-GTP/Total Rap1 ratio is indicated beneath the blots. *p < 0.05, ***p < 0.001. tg: transgenic; wt: wild-type; T: thrombin; U0: U0126; SHP: SHP099.

The above results demonstrate that ERKs and p38 MAPKs promote Tyr504-C3G phosphorylation but not through the activation of its main kinase, Src. This suggests that these MAPKs could regulate C3G by impairing its dephosphorylation. Shp2 (PTPN11, SH-PTP2) is one of the main tyrosine phosphatases that regulate platelet functions.^21^ Shp2 is phosphorylated and inhibited by ERKs in PC12 cells.^24^ Therefore, we determined whether Shp2 is involved in the regulation of Tyr504-C3G phosphorylation mediated by ERKs by pretreating platelets with a selective inhibitor of Shp2, SHP099. As shown in Fig. 6a, SHP099 completely reversed the inhibitory effect of U0126 on thrombin-induced Tyr504-C3G phosphorylation. This result indicates that ERKs stimulate C3G phosphorylation at Tyr504 through inhibiting the phosphatase Shp2, which is responsible for the dephosphorylation of this residue.

### Shp2 colocalizes with C3G and inhibits its GEF activity

To confirm that the Shp2 phosphatase is regulated by ERKs and responsible for the dephosphorylation of C3G, we studied whether Shp2 and pTyr504-C3G colocalize in tgC3G platelets. Manders’ colocalization coefficient analysis revealed that pTyr504-C3G and Shp2 indeed colocalized upon thrombin stimulation (Fig. 6b). This colocalization was maximal in the presence of U0126, a condition in which Shp2 activation is increased, and coincided with increased C3G dephosphorylation. This colocalization was partially lost when platelets were pretreated with SHP099 but recovered when U0126 was also present. These results indicate that the colocalization of pTyr504-C3G and Shp2 depends on the activation of Shp2.

Finally, to verify that Shp2 negatively regulates C3G activation, we analyzed the levels of active Rap1 in the lysates of transgenic platelets treated with thrombin in the presence of U0126 and/or SHP099. Figure 6c shows a slight decrease in Rap1-GTP levels in tgC3G platelets pretreated with U0126 prior to thrombin stimulation, according to the positive effect of ERKs on C3G. In contrast, pretreatment with SHP099 further increased Rap1-GTP levels induced by thrombin, consistent with the negative effect of Shp2 on C3G activity. Moreover, SHP099 reversed the inhibitory effect of U0126 on Rap1-GTP levels. The inhibition of Shp2 did not modify the levels of Rap1-GTP in tgC3GΔCat or wild-type platelets, suggesting that this regulation is specific for C3G and does not apply to other Rap1 GEFs. A slight increase in Rap1-GTP levels was observed in control platelets treated with U0126. This could be explained by the negative regulation of CalDAG-GEFI activity by ERKs, as previously described.^40^ All these results support the notion that Shp2 is the phosphatase regulated by ERKs that dephosphorylates and inhibits C3G.

### C3G participates in TXA_2_ activities in platelets

**Fig. 7:**
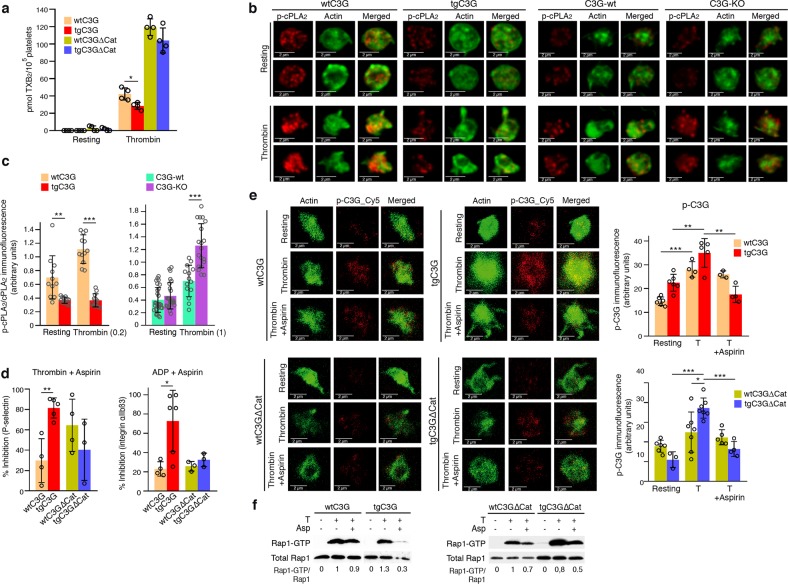
C3G participates in the activities of the TXA2 pathway in platelets. **a** TgC3G expression negatively regulates the production of TXB_2_ induced by thrombin. Platelets from mice of the different genotypes under study were stimulated with thrombin (1 U/ml) for 3.5 min at 37 °C while stirring, and secreted TXA_2_ was determined by detecting its breakdown product, TXB_2_, by LC/MS/MS. The histograms represent the mean ± SD of the amount of TXB_2_ (pmol) per 10^5^ platelets. **b** C3G inhibits cPLA_2_ phosphorylation. Representative immunofluorescence confocal microscopy images of tgC3G and C3G-KO platelets and their corresponding controls treated with 0.2 U or 1 U/ml thrombin, as indicated, and stained with anti-phospho-cPLA_2__Alexa Fluor 568 (red) and phalloidin (green). The images of wtC3G/tgC3G and C3G-wt/C3G-KO platelets correspond to two different experiments, but all images from each experiment were taken at the same exposure time. Bar: 2 μm. **c** The histograms represent the mean ± SD (*n* > 10) of the fluorescence intensity (arbitrary units) of phospho-cPLA_2_ relative to the levels of total cPLA_2_ (Fig. S7a), as quantified by ImageJ. **d** tgC3G and tgC3GΔCat platelets showed different sensitivities to the activity of aspirin. Transgenic platelets and their controls were pretreated with 2 mM aspirin for 5 min and then stimulated for 15 min with 1 U/ml thrombin or 10 μM ADP. The histograms represent the mean ± SD of the percentage of inhibition of P-selectin expression on the surface or activation of integrin αIIbβ3 in platelets treated with thrombin + aspirin or ADP + aspirin, compared to agonist-treated platelets. **e** Left: R**e**presentative immunofluorescence confocal microscopy images of platelets of each genotype under each treatment condition (resting, thrombin treatment, thrombin + aspirin treatment) stained with anti-pTyr504-C3G_Cy5 (red) and phalloidin (green) were taken at the same exposure time. Bar: 2 μm. Right: The histograms represent the mean ± SD of the fluorescence intensity (arbitrary units) of pTyr504-C3G (p-C3G) relative to the phalloidin signal, as quantified by ImageJ. **f** Platelets were pretreated with 2 mM aspirin for 5 min and then stimulated with 0.2 U/ml thrombin for an additional 5 min. Rap1-GTP was isolated by pulldown with GST-RalGDS-RBD and detected by immunoblotting with anti-Rap1 antibodies. Values are relative to the thrombin value in control platelets and were normalized against total Rap1. The Rap1-GTP/Total Rap1 ratio is indicated beneath the blots. *p < 0.05, **p < 0.01, ***p < 0.001. tg: transgenic; wt: wild-type; T: thrombin, Asp: aspirin.

One of the consequences of platelet activation is the production of TXA_2_, a platelet agonist that amplifies platelet responses through its release and binding of specific receptors in the plasma membrane. The above results indicate that C3G participates in the ERK and p38 MAPK pathways, leading to platelet activation and aggregation. Since these pathways are also responsible for the synthesis and secretion of TXA_2_ through the activation of cPLA_2_,^34^ we investigated whether C3G plays a role in the regulation of TXA_2_ synthesis and whether it participates in platelet responses to TXA_2_. HPLC/mass spectrometry analysis of thrombin-stimulated platelets revealed that the levels of released TXB_2_ (the stable form of TXA_2_) were significantly lower in tgC3G platelets than in wtC3G platelets (Fig. 7a). Despite the negative effect of C3G on ERKs and p38 MAPKs described in many cell types,^16,35,36,38,39^ we did not see such effect in platelets. Therefore, we explored whether the thrombin-C3G pathway interferes with TXA_2_ synthesis at the level of cPLA_2_ activation. To do so, we analyzed the phosphorylation of cPLA_2_ at Ser505, which increases its activity, by immunofluorescence.^41^ Indeed, tgC3G platelets showed significantly lower levels of phosphorylated cPLA_2_ than wtC3G platelets, both at rest and upon thrombin stimulation (0.2 U/ml). Accordingly, C3G-KO platelets exhibited higher levels of p-cPLA2 than their wild-type controls in response to 1 U/ml thrombin (Fig. 7b, c and S7a). These results suggest a negative role of C3G in cPLA2 activation, which could explain the decreased production of TXA_2_ by tgC3G platelets.

Next, we evaluated the participation of C3G in TXA_2_-mediated platelet activation and aggregation by analyzing the sensitivities of platelets of the different genotypes to the effects of aspirin. The tgC3G platelets were more sensitive to aspirin-mediated inhibition than the wtC3G platelets (Figs. 7d, S7b, c), and statistically significant decreases in the activation of integrin αIIbβ3 induced by ADP and in the levels of P-selectin present on the surface in response to thrombin were found (Fig. 7d). These results suggest that C3G participates in the activities of TXA_2_ in platelets. In agreement with these results, pretreatment with aspirin abrogated thrombin-induced C3G phosphorylation at Tyr504 (Fig. 7e). The inhibitory effect of aspirin was significantly higher in both types of transgenic platelets, which suggests that TXA_2_ is a mediator of the activation of C3G induced by thrombin. Consequently, Rap1 activity was clearly more sensitive to aspirin in tgC3G platelets than in wtC3G platelets, with no differences in Rap1 activity between tgC3GΔCat platelets and their controls observed (Fig. 7f).

## Discussion

Using transgenic mouse models, our group previously demonstrated that C3G participates in platelet activation and aggregation induced by thrombin, PMA, ADP, and collagen.^3^ In this article, we confirm the implication of C3G in these platelet functions and go beyond through the use of a C3G-knockout model specific for MKs and platelets.

In platelets, thrombin induces the rapid activation of Rap1b, mediated by calcium-CalDAG-GEFI (RasGRP2).^25,42^ This is followed by a second wave of Rap1b activation dependent on PKC in which C3G is the main GEF.^3,8^ The participation of C3G in the PKC-Rap1b pathway is fully supported by the immunofluorescence experiments presented in Fig. 2, which show the total dependence of Tyr504-C3G phosphorylation on PKC. This phosphorylation is very important for full C3G activation.^26^ However, since PKC is a Ser/Thr kinase, it cannot be directly responsible for C3G phosphorylation. Our results indicate that, as previously described,^19^ PKC is involved in the activation of Src, which, in turn, phosphorylates C3G at Tyr504. This is in agreement with the well-known role of Src in C3G phosphorylation in other systems.^18^ Hence, Src serves as the link between PKC and C3G in platelet signaling. These results are consistent with our recent findings showing an increase in the levels of pTyr504-C3G in K562 and HEL cells in response to PMA-induced megakaryocytic differentiation.^2^

It is important to mention that due to the low C3G protein levels present in wild-type platelets, together with the lack of suitable phospho-C3G antibodies for western blotting, it is extremely difficult to analyze C3G phosphorylation in platelets with this technique. Therefore, we performed these analyses using confocal fluorescence techniques, which are much more sensitive than western blotting and provided highly reproducible results in this model.

We previously found that transgenic C3G platelets exhibited greater activation and aggregation in response to ADP than their wild-type controls.^3^ ADP activates Rap1b through two alternative pathways: the P2Y1-Gαq-PLCβ-CalDAG-GEFI and P2Y12-Gαi-PI3K pathways.^10^ Here, we show that C3G preferentially participates in the pathway activated by the ADP receptor P2Y12, which is supported by the significantly increased sensitivity of tgC3G platelets, compared to wtC3G platelets, to the inhibition of integrin αIIbβ3 activation by clopidogrel and wortmannin. This is reinforced by the fact that tgC3GΔCat platelets showed the opposite tendency. Accordingly, the activation of Rap1b induced by thrombin was also more sensitive to the inhibition of P2Y12 and PI3K in tgC3G platelets than in wtC3G platelets. Collectively, these results indicate that C3G participates in the ADP-P2Y12-PI3K pathway leading to Rap1b activation. In addition, our results show the poor contribution of C3G to P2Y1 responses, which is in agreement with the important role of CalDAG-GEFI in the activation of Rap1b by this pathway.^10^

**Fig. 8:**
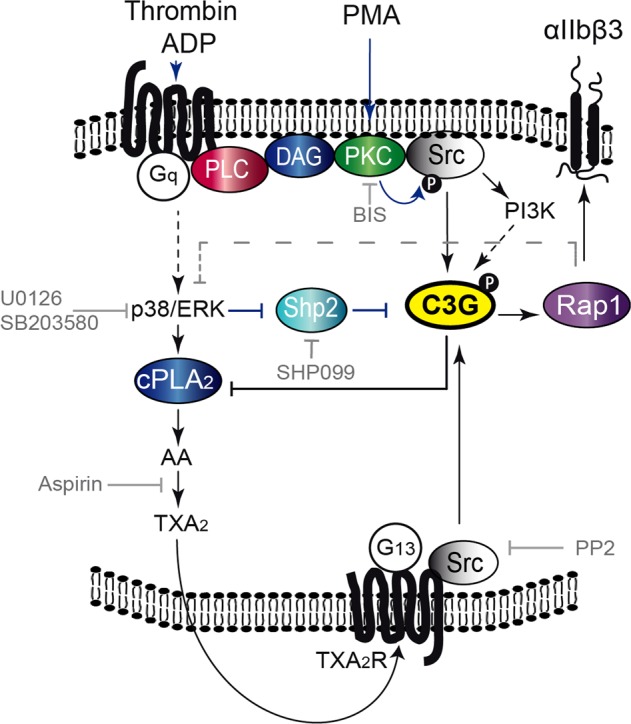
Proposed model for the participation of C3G in platelet signaling. C3G participates in the second wave of Rap1 activation induced by thrombin, which leads to its activation by phosphorylation at Tyr504 through the PKC-Src pathway. C3G phosphorylation is also regulated by ERKs and p38 MAPKs: ERKs inhibit the Shp2 tyrosine phosphatase, allowing C3G phosphorylation, and both MAPKs control the production of TXA_2_. C3G, via Rap1-dependent and -independent mechanisms, regulates TXA_2_ synthesis through a negative feedback loop involving the inhibition of cPLA_2_. PLC: phospholipase C; DAG: diacylglycerol; AA: arachidonic acid; TXA_2_: thromboxane A2; TXA_2_R: TXA_2_ receptor; cPLA_2_: cytosolic phospholipase A_2_; BIS: bisindolylmaleimide, an inhibitor of PKC; PP2: a Src inhibitor; wortmannin: a PI3K inhibitor; SHP099: a Shp2 inhibitor; U0126: an ERK inhibitor; SB203580: a p38α/β MAPK inhibitor; aspirin: a cyclooxygenase inhibitor. Dashed gray lines indicate a hypothetical Rap1-dependent pathway that could regulate the activation of ERKs and p38 MAPKs.

In concordance with most other authors, our results support the participation of ERKs (mainly ERK2) and p38 MAPKs in platelet functions.^6,13^ Hence, both are phosphorylated in response to thrombin, and their inhibition decreases the activation and aggregation of wild-type platelets. The activation of ERKs by thrombin was determined at a high thrombin concentration (1 U/ml), a condition in which thrombin preferentially signals through PKC independently of ADP.^17,43^ This would explain the lack of the inhibitory effect of clopidogrel or 2-MeSAMP on thrombin-induced ERK phosphorylation. Transgenic C3G platelets were consistently more sensitive to ERK and p38α/β inhibition, suggesting the participation of C3G in signaling pathways mediated by these MAPKs. In fact, thrombin-induced C3G phosphorylation at Ty504 was drastically inhibited by U0126 and SB203580, providing an explanation for the increased sensitivity of tgC3G platelet activation and aggregation to both inhibitors. These results suggest that C3G is positively regulated by these MAPKs. A functional relationship, both positive and negative, between C3G and these MAPKs has been well documented.^35–39^ In platelets, the contribution of C3G to the transient activation of ERKs and p38 MAPKs appears to be marginal. This is in agreement with our results in K562 cells, as well as those in other systems, showing that C3G-Rap1 participates in the sustained (and not transient) activation of ERKs.^44^ Instead, ERKs and p38 MAPKs are involved in pathways leading to C3G phosphorylation. Specifically, ERKs promote C3G phosphorylation through inhibition of the phosphatase Sph2, which is responsible for the dephosphorylation of C3G at Tyr504 (Fig. 8). In platelets, Shp2 inhibits pathways responsible for late outside-in signaling (e.g., the phosphorylation of Akt and ERKs) but has no effects on pathways responsible for early outside-in signaling (e.g., those mediated by the thrombin receptor), like Src phosphorylation.^22^ However, the inhibitory effect of Shp2 by ERKs had not been previously described in platelets, although the in vitro phosphorylation of Shp2 by ERKs was shown to inhibit its phosphatase activity.^24^ The regulation of C3G by the ERK-Shp2 axis is supported by the observation that C3G and Shp2 colocalize and that this colocalization is maximal under conditions of ERK inhibition. In addition, Shp2 inhibition reversed the decrease in Rap1-GTP levels produced by U0126. Taken together, these results suggest the existence of a positive regulatory mechanism mediated by ERKs that, through the inhibition of Shp2, enhances the phosphorylation of C3G induced by thrombin. Interestingly, ERKs inhibit CalDAG-GEFI,^40^ which raises the idea that ERKs can positively or negatively regulate the activity of Rap1b through the modulation of its GEFs.

Our results also showed that ERKs are activated by PKC in platelets, in agreement with the role of PKC in the activation of ERKs during TXA_2_ synthesis.^34^ It has been suggested that TXA_2_, acting through its receptor, TXA_2_R, contributes to Rap1b activation in response to ADP and collagen but not thrombin.^15^ In contrast, our results are consistent with the existence of a TXA_2_-C3G-Rap1 pathway activated by thrombin, as suggested by (i) the greater sensitivity of tgC3G platelets to aspirin during thrombin-induced activation and aggregation; (ii) the inhibitory effect of aspirin on the phosphorylation of C3G induced by thrombin, which was more evident in tgC3G platelets; and (iii) the inhibitory effect of aspirin on C3G-mediated Rap1 activation induced by thrombin. Moreover, C3G might reduce the levels of TXA_2_ through modulating the activity of cPLA_2_, as also demonstrated in our C3G-KO model. All these results lead us to propose a model in which MAPKs, in response to thrombin, regulate the activation of Rap1 through a TXA_2_-TXA_2_R-C3G axis (Fig. 8). Further studies should be aimed at discerning the precise mechanism by which C3G modulates cPLA_2_.

In wild-type platelets, Rap1 activity was hardly affected by the tested inhibitors (wortmannin, 2-MeSAMP, aspirin, U0126 or SHP099). This suggests that Rap1 activity is tightly modulated by compensatory mechanisms, highlighting its relevant role in platelet physiology. In contrast, tgC3G platelets were very sensitive to these inhibitors, indicating that the overexpression of C3G made platelets more dependent on this RapGEF. However, the data derived from our knockout model confirmed the important role of endogenous C3G in platelet activation and aggregation based on its GEF activity for Rap1, especially that in response to PMA and thrombin at a high concentration.

In summary, endogenous C3G plays a physiological role in platelets by promoting Rap1 activation. In addition, the transgenic expression of C3G enhances thrombin- and ADP-mediated platelet activation and aggregation through its participation in the PKC-Src, P2Y12-PI3K, and p38/ERKs-TXA_2_ signaling pathways. The activation of C3G by its phosphorylation at Tyr504 is regulated by PKC-Src and two mechanisms involving MAPKs: one that acts through inhibition of the Sph2 PTP and another that acts through the production of TXA_2_. Therefore, C3G is a Rap1 GEF with specific functions that are complementary to those of CalDAG-GEFI, allowing the tight modulation of Rap1 activity in platelets.

Hence, C3G may play a relevant role in physiological and/or pathological processes involving platelets, such as cancer or cardiovascular diseases, and may represent a new biomarker and/or therapeutic target in platelets.

## Materials and methods

### Mouse models

A conditional knockout mouse model for *Rapgef1* in which exons 17–21 were flanked by LoxP sites was generated as described.^45^ To delete C3G from MKs and platelets, *Rapgef1*^flox/flox^ mice were crossed with PF4-Cre^+/−^ transgenic mice,^46^ generating *Rapgef1*^flox/flox^; PF4-Cre^+/−^ (C3G-KO) and *Rapgef1*^flox/flox^; PF4-Cre^−/−^ (C3G-wt) offspring. The offspring were genotyped by PCR with the following primers: conditional and wild-type *Rapgef1* alleles: C3G-KO-LoxF 5′-AGCCTGTTGGCAAGTTTGG-3′ and C3G-KO-LoxR 5′-CTGATGGAGAACCTAGCTGTGG-3′; PF4-Cre and wild-type alleles: PF4-prom-F 5′-CCC ATA CAG CAT ACC TTT TG-3′, PF4-prom-Cre-R 5′-TGC ACA GTC AGC AGG TT-3′ and PF4-prom-WT2-R 5′-TTA CAG CAT CGC CTT CCC CT-3′. Deletion of the flanked exons in MK DNA was monitored by PCR with the primers C3G-KO-LoxF and C3G-int21R (5′-GGACTGGAGCATCTTTCAG-3′) (Fig. S1a and b).

The transgenic mice models used in this work have been previously described.^2–4^ The human transgenes C3G (full-length) and C3GΔCat (deletion in the catalytic region) were expressed under the control of the platelet-specific PF4 gene promoter. Transgenic C3G (tgC3G) lines 2C1 and 6A6 and transgenic C3GΔCat (tgC3GΔCat) line 8A3 were used.

WtC3G and wtC3GΔCat mice were the control mice for tgC3G and tgC3GΔCat mice, respectively, while C3G-wt mice were the control mice for the C3G-KO model.

All mice used in these studies were 10–14 weeks old, unless otherwise specified.

### Platelet purification

Blood (0.5–1 ml) was collected by cardiac puncture from isoflurane (2%)-anesthetized mice into citrate tubes (Sarstedt, Germany) and used to prepare platelet-rich plasma (PRP) by double centrifugation at 100 × *g* for 4 and 5 min. Platelets were pelleted by centrifugation at 1300 × *g* for 5 min and resuspended in Tyrode-Hepes buffer (134 mM NaCl, 0.34 mM Na_2_HPO_4_, 2.9 mM KCl, 12 mM NaHCO_3_, 20 mM Hepes (pH 7.0), 5 mM glucose) at 2 × 10^8^ platelets/ml.

### Purification of MK DNA from the bone marrow (BM) and spleen

BM cells were obtained from the femora and tibiae of mice by flushing with PBS and centrifuged for 5 min at 1300 × *g*. Erythrocytes were lysed with red cell blood lysis buffer (RCB, 0.155 M NH_4_Cl, 10 mM KHCO_3_, 10 mM EDTA, pH 7.4) for 15 min at 4 °C. MKs were isolated by BSA density gradient created by layering 1, 3, and 5% BSA in a Falcon tube. BM cells were gently introduced into the gradient, and after 1 h, MKs were collected from the bottom.

The spleen was digested with 1 mg/ml collagenase and dispase for 45 min at 37 °C while stirring. Cells were filtered using a 70 μm nylon cell strainer (Falcon) and washed with cold PBS. Erythrocytes were lysed by incubation with RCB for 15 min at 4 °C. Cells were incubated with 1:50 anti-CD41-APC (eBioscience (ThermoFisher Scientific), 17-0411) and anti-CD61-PE (eBioscience, 12-0611) antibodies for 15 min at 4 °C. Double-labeled cells (MKs) were sorted using a BD FACSAria II cytometer.

Genomic DNA was purified from lysed MKs by precipitation with 5 M NaCl and ethanol, as described.^47^

### Real-time PCR

cDNA was obtained from MKs and platelets as described.^3^ C3G expression was monitored by qPCR using SYBR Green master mix (Biotools, Spain) and the following primers: N-terminus: 190F 5′-GTGAGCAAAGAGGCAAGAGA-3′ and 288R 5′-CACAGCACTGGTGGACATAA-3′ and C-terminus: 2540F 5′- ATTTCCACAGCCACGAGATAG-3′ and 2654R 5′-CTCTTCTCCTCATTCTGCTCTT-3. β-Actin, which was used as a housekeeping gene, was amplified with the oligos F 5′-TAGACTTCGAGCAGGAGATGG-3′ and R 5′- CAAGAAGGAAGGCTGGAAAG-3′. Primers were designed with the *PrimerQuest Tool* (Integrated DNA Technologies, IA, USA), and Tm values were calculated with *Oligo Calc: Oligonucleotide Properties Calculator* (http://biotools.nubic.northwestern.edu/OligoCalc.html). Data were analyzed using the 2^-ΔΔ CT^ method.^48^

### Tail-bleeding assay

A 5 mm section of the tail tip from each anesthetized mouse was transected, and the tail was immersed in 37 °C PBS, following which the time until blood flow ceased was noted. The experiment was terminated after 8 min. Measurements were made in 3-week-old mice at weaning (before genotyping).

### Model of pulmonary thromboembolism

C3G-KO and C3G-wt mice were anesthetized with a mixture of ketamine and valium (100 mg/kg of mouse body weight and 5 mg/kg of mouse body weight, respectively, in 0.9% NaCl), and the jugular vein was exposed. A mixture of 600 μg of collagen (type I, Sigma, MO, USA) and 60 μg of norepinephrine (Sigma)/kg of mouse body weight (lethal dose) or 150 μg of collagen and 15 μg of norepinephrine/kg of mouse body weight (sublethal dose) was injected into the jugular vein. At the lethal dose, acute pulmonary thromboembolism occurred, and the mice died by asphyxiation, usually within 10 min. Therefore, the time to death was monitored. Mice that were still alive after 20 min were considered survivors. The sublethal dose induced thrombogenesis, which resulted in thrombocytopenia. This was measured as the decrease in platelet count. Prior to the injection of agonists, a small sample of blood (100–200 μl) was extracted from the contralateral jugular vein into an EDTA tube, and the number of platelets was determined by flow cytometry. Blood withdrawal was repeated 4 min after the injection of collagen/norepinephrine. The difference in platelet count is expressed as the percentage of platelet reduction. The formation of thrombi in the lungs was verified by immunohistochemical analysis of lung sections stained with hematoxylin/eosin. The samples were analyzed with the double-blind method so that we did not know the genotype of each mouse until the end of the histopathological analysis.

### Platelet count by flow cytometry

Fifteen microliters of EDTA-anticoagulated blood was diluted in 500 μl of Tyrode-Hepes buffer. Thirty microliters of this dilution was stained with 1 μl of anti-mouse CD41-FITC (MWReg30 clone, eBioscience) for 15 min and then diluted again to 1 ml. Platelets from 50 μl of this last dilution were measured on a BD Accuri™ C6 flow cytometer.

### Agonist and antagonists for in vitro studies

Platelets were stimulated with 0.2, 0.5, or 1 U/ml thrombin; 0.2, 2, 5, or 10 μM PMA; or 10 μM ADP after treatment with the following inhibitors for 5 min: bisindolylmaleimide (5 μM), clopidogrel (100 μM), 2-MeSAMP (50 μM), MRS2179 (20 μM for immunoblotting, 100 μM for platelet activation and aggregation), U0126 (20 μM), SB203580 (20 μM), wortmannin (100 nM), aspirin (2 mM), PP2 (10 μM) or SHP099 (20 μM).

### Confocal immunofluorescence microscopy

Platelets from four mice of each genotype were pooled and activated as indicated. After pretreatment with inhibitors for 5 min, the platelets were stimulated with 0.5 U/ml thrombin or 2 μM PMA for 1 min at 37 °C while stirring (900 rpm). The platelets were fixed for 15 min in 4% formaldehyde, placed onto poly-l-lysine-coated coverslips and allowed to settle for 50 min. Attached platelets were permeabilized with 0.2% Triton X-100 and blocked overnight in PBS with 1% BSA. The platelets were incubated with the following primary antibodies at RT for 2 h: Oregon Green® 514 phalloidin (Thermo Fisher Scientific, MA, USA); C3G-1008 antiserum^49^; Src (phospho Y418, Ab4816) from Abcam (UK); p-C3G (Tyr504, sc-12926), SH-PTP2 (B-1, sc-7384), and cPLA_2_ (4-4B-3C, sc-454) from Santa Cruz Biotechnology, Inc. (CA, USA); and phospho-cPLA_2_ (Ser 505, #2831) from Cell Signaling Technology Inc. (MA, USA). This was followed by incubation (1 h at RT) with the following secondary antibodies as indicated: Cy3-conjugated goat anti-rabbit (Abcam); Cy5-conjugated goat anti-mouse (Jackson ImmunoResearch Europe Ltd, UK); and Alexa Fluor 568 goat anti-rabbit or Alexa Fluor 647 goat anti-mouse (Thermo Fisher Scientific). Images were obtained at the same exposure time with Zeiss LSM 515 and Leica TCS SP8 confocal microscopes, and pictures were processed using Zen 2.1 imaging software and LSM Image Browser. Colocalization was determined by Manders’ colocalization coefficient (M) analysis using ImageJ software with the JACoP plugin as described.^50^

### Flow cytometric analysis of platelet activation and aggregation

Platelet activation was determined by flow cytometry via measuring the expression of P-selectin on the surface (with FITC rat anti-mouse CD62P antibody from BD Biosciences, CA, USA) as an indicator of platelet degranulation or the high-affinity conformation of the integrin αIIbβ3 (with Alexa Fluor® 488-labeled fibrinogen from Thermo Fisher Scientific or PE-labeled rat anti-mouse αIIbβ3 [JON/A clone] antibody from Emfret Analytics, Germany) as described previously.^3^

Platelet aggregation was determined as previously described.^51^ Briefly, 50 μl of citrate-anticoagulated blood was washed in Tyrode-Hepes buffer and divided into two populations. Each population was labeled with either anti-CD9-FITC or anti-CD9-PE for 15 min. After centrifugation to remove the excess antibodies, the blood was suspended in Tyrode-Hepes, and both populations were mixed. The mixture was incubated with the corresponding inhibitors for 2 min at RT, and aggregation was induced by the addition of 1 U/ml thrombin, 10 μM PMA or 10 μM ADP. The reaction was maintained at 37 °C with stirring at 900 rpm and stopped by the addition of a 9× volume of 0.5% formaldehyde in PBS. Aggregate formation was measured as the percentage of double labeling events.

For both types of analysis, each data point corresponds to one animal.

### Western blot analysis

Platelets suspended in Tyrode-Hepes buffer were lysed in the same volume of 2× modified RIPA buffer (100 mM Tris (pH 7.5), 400 mM NaCl, 5 mM MgCl_2_, 2% NP-40, 2% glycerol, 2 mM Na_3_VO_4_, 50 mM NaF, 2 mM PMSF, 2 μM aprotinin, 2 μM leupeptin). Protein lysates were resolved by SDS-PAGE and transferred to PVDF membranes that were blotted using the following antibodies: anti-p-ERK (E-4) (sc-7383), anti-ERK 1 (sc-94), and anti-p38α (sc-535) from Santa Cruz Biotechnology, Inc.; anti-phospho-p38 MAP kinase Thr180/Tyr182 (#9211) from Cell Signaling Technology Inc.; and anti-Rap1 (#610196) from BD Biosciences.

### TXA2 measurement

Platelets were stimulated with 1 U/ml thrombin for 3.5 min while shaking (900 rpm). Then, the platelets were pelleted, and the supernatants were collected. The same volume of methanol was added to the supernatants, and the vials were purged with nitrogen prior to snap-freezing. Secreted TXA_2_ was measured by liquid chromatography coupled to tandem mass spectrometry (LC/MS/MS) as previously described.^52^ TXA_2_ was detected via detection of its breakdown product, TXB_2_.

### Rap1 activation assay

The activation of Rap1 was evaluated by pulldown assay as described previously^3^ using GST-RalGDS RBD immobilized on glutathione-sepharose, which binds specifically and selectively to the GTP-bound form of Rap1b. Platelets were pretreated with different inhibitors for 5 min prior to stimulation with thrombin or PMA for 1 or 5 min.

### Calculation of sensitivity to inhibitors

Platelet activation and aggregation in platelets treated with thrombin, PMA or ADP in the presence or absence of different inhibitors were monitored by flow cytometry as described above. The effects of the inhibitors on platelet activation and aggregation were calculated as the percentage of inhibition with the following formula:

$$\left( {1 - \frac{\% \ {\mathrm{activation}}\ {\mathrm{or}}\ {\mathrm{aggregation}}\ {\mathrm{with}}\ {\mathrm{agonist}} + {\mathrm{inhibitor}}}{\% \ {\mathrm{activation}}\ {\mathrm{or}}\ {\mathrm{aggregation}}\ {\mathrm{with}}\ {\mathrm{agonist}}}} \right) \times 100$$

### Statistical analysis

Data are represented as the mean ± SD (standard deviation) or SEM (standard error of the mean) as indicated. The Kolmogorov-Smirnov test was performed to determine if data fit a normal distribution. To compare two experimental groups for which the data were normally distributed, the unpaired Student’s t-test was carried out. The nonparametric Mann–Whitney *U*-test was carried when the data were not normally distributed. Differences were considered significant when *p* < 0.05. The effect of lethal collagen/epinephrine injection on mouse survival was evaluated using Kaplan–Meier Plotter and the log-rank test (SigmaPlot 12 software).

## Supplementary information

Supplemental material

## References

[1] Stefanini L, Bergmeier W (2018). RAP GTPases and platelet integrin signaling. Platelets.

[2] Ortiz-Rivero S (2018). C3G, through its GEF activity, induces megakaryocytic differentiation and proplatelet formation. Cell Commun. Signal..

[3] Gutiérrez-Herrero S (2012). C3G transgenic mouse models with specific expression in platelets reveal a new role for C3G in platelet clotting through its GEF activity. Biochim. Biophys. Acta Mol. Cell. Res..

[4] Martín-Granado V (2017). C3G promotes a selective release of angiogenic factors from activated mouse platelets to regulate angiogenesis and tumor metastasis. Oncotarget.

[5] Benz PM (2016). Vasodilator-stimulated phosphoprotein (VASP)-dependent and -independent pathways regulate thrombin-induced activation of Rap1b in platelets. Cell Commun. Signal..

[6] Li Z, Delaney MK, O´Brien KA, Du X (2010). Signaling during platelet adhesion and activation. Arterioscler. Thromb. Vasc. Biol..

[7] Brass LF, Tomaiuolo M, Stalker TJ (2013). Harnessing the platelet signaling network to produce an optimal hemostatic response. Hematol. Oncol. Clin. North Am..

[8] Franke B (2000). Sequential regulation of the small GTPase Rap1 in human platelets. Mol. Cell. Biol..

[9] Lova P, Paganini S, Sinigaglia F, Balduini C, Torti M (2002). A Gi-dependent pathway is required for activation of the small GTPase Rap1B in human platelets. J. Biol. Chem..

[10] Woulfe D, Jiang H, Mortensen R, Yang J, Brass LF (2002). Activation of Rap1B by G(i) family members in platelets. J. Biol. Chem..

[11] Smyth SS (2009). G-protein-coupled receptors as signaling targets for antiplatelet therapy. Arterioscler. Thromb. Vasc. Biol..

[12] Shankar H, Garcia A, Prabhakar J, Kim S, Kunapuli SP (2006). P2Y12 receptor-mediated potentiation of thrombin-induced thromboxane A2 generation in platelets occurs through regulation of Erk1/2 activation. J. Thromb. Haemost..

[13] Mazharian A (2007). Protease-activating receptor-4 induces full platelet spreading on a fibrinogen matrix: involvement of ERK2 and p38 and Ca2+ mobilization. J. Biol. Chem..

[14] McNicol A, Jackson EC (2003). Inhibition of the MEK/ERK pathway has no effect on agonist-induced aggregation of human platelets. Biochem. Pharmacol..

[15] Zhang G (2011). Distinct roles for Rap1b protein in platelet secretion and integrin αIIbβ3 outside-in signaling. J. Biol. Chem..

[16] Stork PJ, Dillon TJ (2005). Multiple roles of Rap1 in hematopoietic cells: complementary versus antagonistic functions. Blood.

[17] Nadal-Wollbold F (2002). Platelet ERK2 activation by thrombin is dependent on calcium and conventional protein kinases C but not Raf-1 or B-Raf. FEBS Lett..

[18] Radha V, Mitra A, Dayma K, Sasikumar K (2011). Signalling to actin: role of C3G, a multitasking guanine-nucleotide-exchange factor. Biosci. Rep..

[19] Senis YA, Mazharian A, Mori J (2014). Src family kinases: at the forefront of platelet activation. Blood.

[20] Lannutti BJ, Minear J, Blake N, Drachman JG (2006). Increased megakaryocytopoiesis in Lyn-deficient mice. Oncogene.

[21] Mazharian A (2013). Megakaryocyte-specific deletion of the protein-tyrosine phosphatases Shp1 and Shp2 causes abnormal megakaryocyte development, platelet production, and function. Blood.

[22] Hu M (2019). Platelet Shp2 negatively regulates thrombus stability under high shear stress. J. Thromb. Haemost..

[23] Pao LI, Badour K, Siminovitch KA, Neel BG (2007). Nonreceptor protein-tyrosine phosphatases in immune cell signaling. Annu. Rev. Immunol..

[24] Peraldi P, Zhao Z, Filloux C, Fischer EH, Van Obberghen E (1994). Protein-tyrosine-phosphatase 2C is phosphorylated and inhibited by 44-kDa mitogen-activated protein kinase. Proc. Natl Acad. Sci. USA.

[25] Crittenden JR (2004). CalDAG-GEFI integrates signaling for platelet aggregation and thrombus formation. Nat. Med..

[26] Ichiba T (1999). Activation of C3G guanine nucleotide exchange factor for Rap1 by phosphorylation of tyrosine 504. J. Biol. Chem..

[27] Shivakrupa R, Radha V, Sudhakar C, Swarup G (2003). Physical and functional interaction between Hck tyrosine kinase and guanine nucleotide exchange factor C3G results in apoptosis, which is independent of C3G catalytic domain. J. Biol. Chem..

[28] Radha V, Rajanna A, Swarup G (2004). Phosphorylated guanine nucleotide exchange factor C3G, induced by pervanadate and Src family kinases localizes to the Golgi and subcortical actin cytoskeleton. BMC Cell Biol..

[29] Mitra A, Radha V (2010). F-actin-binding domain of c-Abl regulates localized phosphorylation of C3G: role of C3G in c-Abl-mediated cell death. Oncogene.

[30] Xu XR (2016). Platelets are versatile cells: New discoveries in hemostasis, thrombosis, immune responses, tumor metastasis and beyond. Crit. Rev. Clin. Lab. Sci..

[31] Li Z, Xi X, Du X (2001). A mitogen-activated protein kinase-dependent signaling pathway in the activation of platelet integrin alpha IIbbeta3. J. Biol. Chem..

[32] Sakurai, K. et al. Role of p38 mitogen-activated protein kinase in thrombus formation. *J. Recept. Signal Transduct. Res*. **24**, 283–296 (2004). 10.1081/rrs-200040324 15648447

[33] Roger S (2004). Costimulation of the Gi-coupled ADP receptor and the Gq-coupled TXA2 receptor is required for ERK2 activation in collagen-induced platelet aggregation. FEBS Lett..

[34] Yacoub D (2006). Essential role of protein kinase Cdelta in platelet signaling, alpha(IIb)beta(3) activation, and thromboxane A(2) release. J. Biol. Chem..

[35] Guerrero C, Martin-Encabo S, Fernandez-Medarde A, Santos E (2004). C3G-mediated suppression of oncogene-induced focus formation in fibroblasts involves inhibition of ERK activation, cyclin A expression and alterations of anchorage-independent growth. Oncogene.

[36] Gutiérrez-Uzquiza A (2010). C3G down-regulates p38 MAPK activity in response to stress by Rap-1 independent mechanisms: Involvement in cell death. Cell. Signal..

[37] Maia V (2013). C3G forms complexes with Bcr-Abl and p38alpha MAPK at the focal adhesions in chronic myeloid leukemia cells: implication in the regulation of leukemic cell adhesion. Cell Commun. Signal..

[38] Maia V (2009). C3G silencing enhances STI-571-induced apoptosis in CML cells through p38 MAPK activation, but it antagonizes STI-571 inhibitory effect on survival. Cell. Signal..

[39] Priego N (2016). C3G knock-down enhances migration and invasion by increasing Rap1-mediated p38α activation, while it impairs tumor growth through p38α-independent mechanisms. Oncotarget.

[40] Ren J, Cook AA, Bergmeier W, Sondek J (2016). A negative-feedback loop regulating ERK1/2 activation and mediated by RasGPR2 phosphorylation. Biochem. Biophys. Res. Commun..

[41] Borsch-Haubold AG (1999). Phosphorylation of cytosolic phospholipase A2 in platelets is mediated by multiple stress-activated protein kinase pathways. Eur. J. Biochem..

[42] Franke B, Akkerman JW, Bos JL (1997). Rapid Ca2+-mediated activation of Rap1 in human platelets. EMBO J..

[43] Falker K, Lange D, Presek P (2004). ADP secretion and subsequent P2Y12 receptor signalling play a crucial role in thrombin-induced ERK2 activation in human platelets. Thromb. Haemost..

[44] Li Y, Dillon TJ, Takahashi M, Earley KT, Stork PJS (2016). PKA-independent Ras activation cooperates with Rap1 to mediate activation of ERKs by cAMP. J. Biol. Chem..

[45] Shah B (2016). C3G/Rapgef1 is required in multipolar neurons for the transition to a bipolar morphology during cortical development. PLoS ONE.

[46] Hurtado B (2018). Thrombocytopenia-associated mutations in Ser/Thr kinase MASTL deregulate actin cytoskeletal dynamics in platelets. J. Clin. Invest.

[47] Miller SA, Dykes DD, Polesky HF (1988). A simple salting out procedure for extracting DNA from human nucleated cells. Nucleic Acids Res..

[48] Livak KJ, Schmittgen TD (2001). Analysis of relative gene expression data using real-time quantitative PCR and the 2(-Delta Delta C(T)) Method. Methods.

[49] Guerrero C (1998). Transformation suppressor activity of C3G is independent of its CDC25-homology domain. Oncogene.

[50] Peters CG, Michelson AD, Flaumenhaft R (2012). Granule exocytosis is required for platelet spreading: differential sorting of α-granules expressing VAMP-7. Blood.

[51] De Cuyper IM (2013). A novel flow cytometry-based platelet aggregation assay. Blood.

[52] Gil-de-Gomez L (2014). Cytosolic group IVA and calcium-independent group VIA phospholipase A2s act on distinct phospholipid pools in zymosan-stimulated mouse peritoneal macrophages. J. Immunol..

