# Supplementary material for: C3G contributes to platelet activation and aggregation by regulating major signaling pathways
Source: Signal Transduct Target Ther. 2020 Apr 1;5:29. doi: 10.1038/s41392-020-0119-9 (PMC7109025; doi:10.1038/s41392-020-0119-9)
Supplement: Supplementary file 1 — Supplemental material [file 41392_2020_119_MOESM1_ESM.docx]

Supplementary Information

This file contains supplementary figures S1-S7.

C3G contributes to platelet activation and aggregation by regulating major signaling pathways

Sara Gutiérrez-Herrero^1^, Cristina Fernández-Infante^1.2†^, Luis Hernández-Cano^1,2†^, Sara Ortiz-Rivero^1^, Carlos Guijas^3,4^, Víctor Martín-Granado^1^, José Ramón González-Porras^2,5^, Jesús Balsinde^3,4^, Almudena Porras^6^*, Carmen Guerrero^1,2,7^*

^1^Instituto de Biología Molecular y Celular del Cáncer (IMBCC), University of Salamanca-CSIC.

^2^Instituto de Investigación Biomédica de Salamanca (IBSAL), Salamanca, Spain.

^3^Instituto de Biología y Genética Molecular (IBGM), Consejo Superior de Investigaciones Científicas (CSIC), University of Valladolid, Valladolid, Spain.

^4^Centro de Investigación Biomédica en Red de Diabetes y Enfermedades Metabólicas Asociadas (CIBERDEM), Madrid, Spain.

^5^Departamento de Hematología, Hospital Universitario de Salamanca (HUS).

^6^Departamento de Bioquímica y Biología Molecular, Facultad de Farmacia, Complutense University of Madrid. Instituto de Investigación Sanitaria del Hospital Clínico San Carlos (IdISSC), Madrid, Spain.

^7^Departamento de Medicina, University of Salamanca, Salamanca, Spain.

*Correspondence: C. Guerrero, Centro de Investigación del Cáncer, Campus Unamuno s/n, Salamanca, Spain. Tel.: +34 923294801; fax.: +34 923294795; e-mail: [cguerrero@usal.es](mailto:cguerrero@usal.es). Cocorrespondence: A. Porras, Departamento de Bioquímica y Biología Molecular, Facultad de Farmacia, UCM, Ciudad Universitaria; IdISSC, Madrid, Spain. Tel.: +34 913941627; fax: +34 913941779; e-mail: [maporras@ucm.es](mailto:maporras@farm.ucm.es)

† These authors contributed equally

Figure S1

**
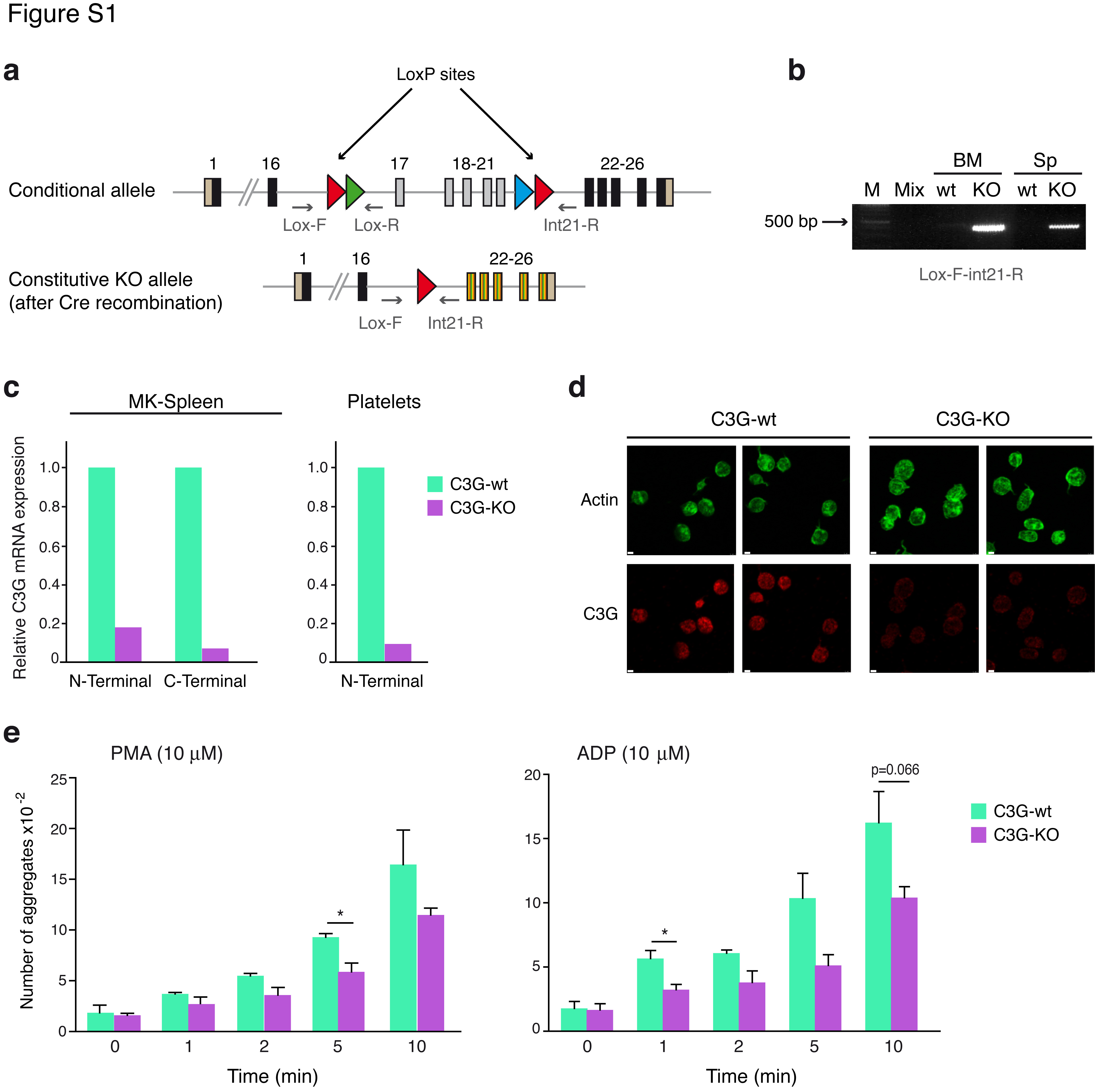
**

**Figure S1. Characterization of the *Rapgef1*^flox/flox^;PF4-Cre^+/-^ (C3G-KO) mouse model in megakaryocytes and platelets.** (**a**) Schematic representation of the targeting strategy used to generate the *Rapgef1*^flox/flox^ conditional knockout, based on (*55*). See this reference for details. Cre-mediated recombination of exons 17-21 (which include part of the catalytic domain), accompanied by a frame-shift from exon 16, result in the inactivation of C3G. (**b**) PCR amplification, using primers Lox-F and int21R, of the genomic DNA of megakaryocytes obtained from the bone marrow (BM) and spleen (Sp) of *Rapgef1*^flox/flox^;PF4-Cre^+/-^ (KO) and control *Rapgef1*^flox/flox^;PF4-Cre^-/-^ (wt) mice. The PCR mix was used as a negative control (Mix). M: Thermo Scientific™ GeneRuler™ 1kb Plus DNA Ladder. As expected, amplification of the ≈400 bp predicted band only occurred in the C3G-KO genomic DNA. (**c**) RT-qPCR analysis of C3G mRNA of spleen megakaryocytes and platelets from C3G-KO and C3G-wt mice. N-terminal amplicon: n190-n288; C-terminal amplicon: n2540-n2654 (NM_054050.2). (**d**) Representative immunofluorescence confocal microscopy images of C3G-KO and C3G-wt platelets stained with phalloidin and antibodies to C3G (rabbit antiserum #1008 (*59*)). Bar: 1 μm. (**e**) C3G-KO platelets show impaired aggregation. Histograms represent the mean ± SEM of the number of platelet aggregates formed upon stimulation with 10 μM PMA (n=3 wt, 4 KO mice) or 10 μM ADP + Alexa Fluor® 488-conjugated fibrinogen for the indicated time periods (n=2 wt, 3 KO mice).

**Figure S2**


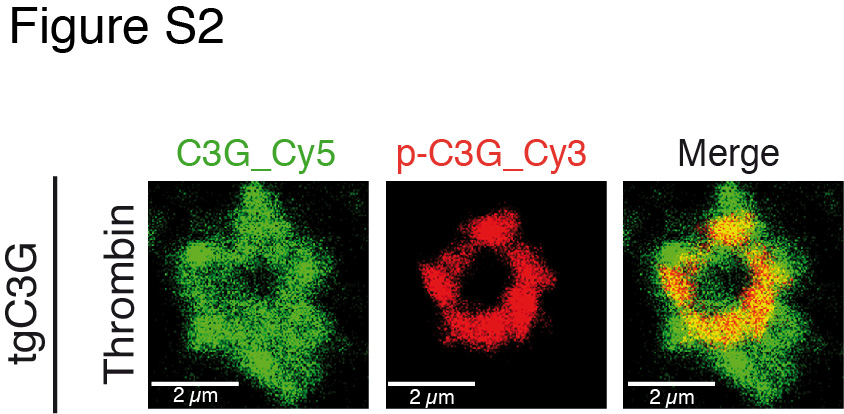


**Figure S2. Colocalization of total C3G and C3G phosphorylated at Tyr504.** Representative immunofluorescence confocal microscopy images of tgC3G platelets showing colocalization of C3G and pTyr504-C3G signals. Platelets were stimulated with 0.5 U thrombin and labeled with anti-C3G antiserum #1008_Cy5 and anti-pTyr504-C3G_Cy3. Images were taken at the same exposure time with a confocal Leica TCS SP8. Bar: 2 μm.

**Figure S3**


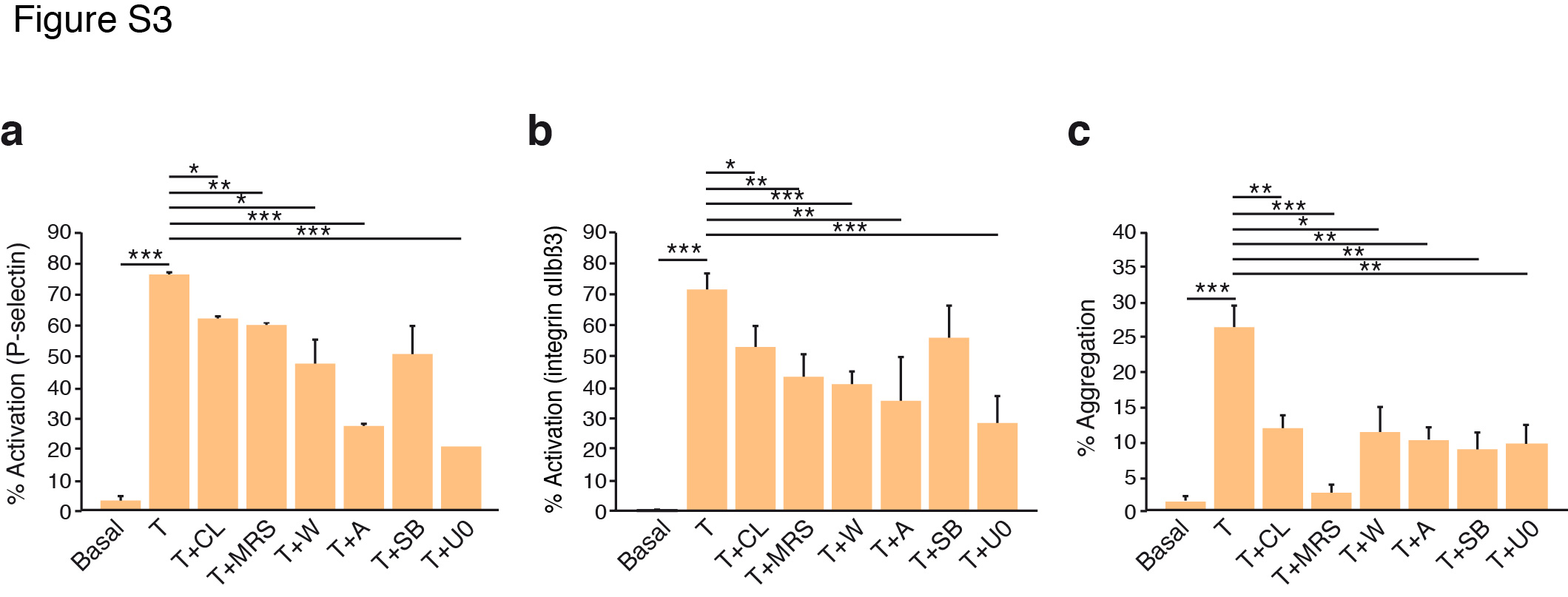


**Figure S3. Effect of inhibitors of platelet signaling pathways on platelet activation and aggregation.** Platelets from wild type mice were pretreated with: 100 μM clopidogrel (CL), 100 μM MRS2179 (MRS), 100 nM wortmannin (W), 2 mM aspirin (A), 20 μM SB203580 (SB) or 20 μM U0126 (U0) for 5 min, and then stimulated with thrombin (1 U/ml) for 15 min (**a, b**) or for 5 min (**c**). The histograms represent the mean ± SEM of the percentage of: (**a**) platelets with P-selectin on the surface (n>6); (**b**) platelets with activated integrin αIIbβ3 (n>8); (**c**) aggregated platelets (n>4). *p<0.05, **p<0.01, ***p<0.001.

**Figure S4**


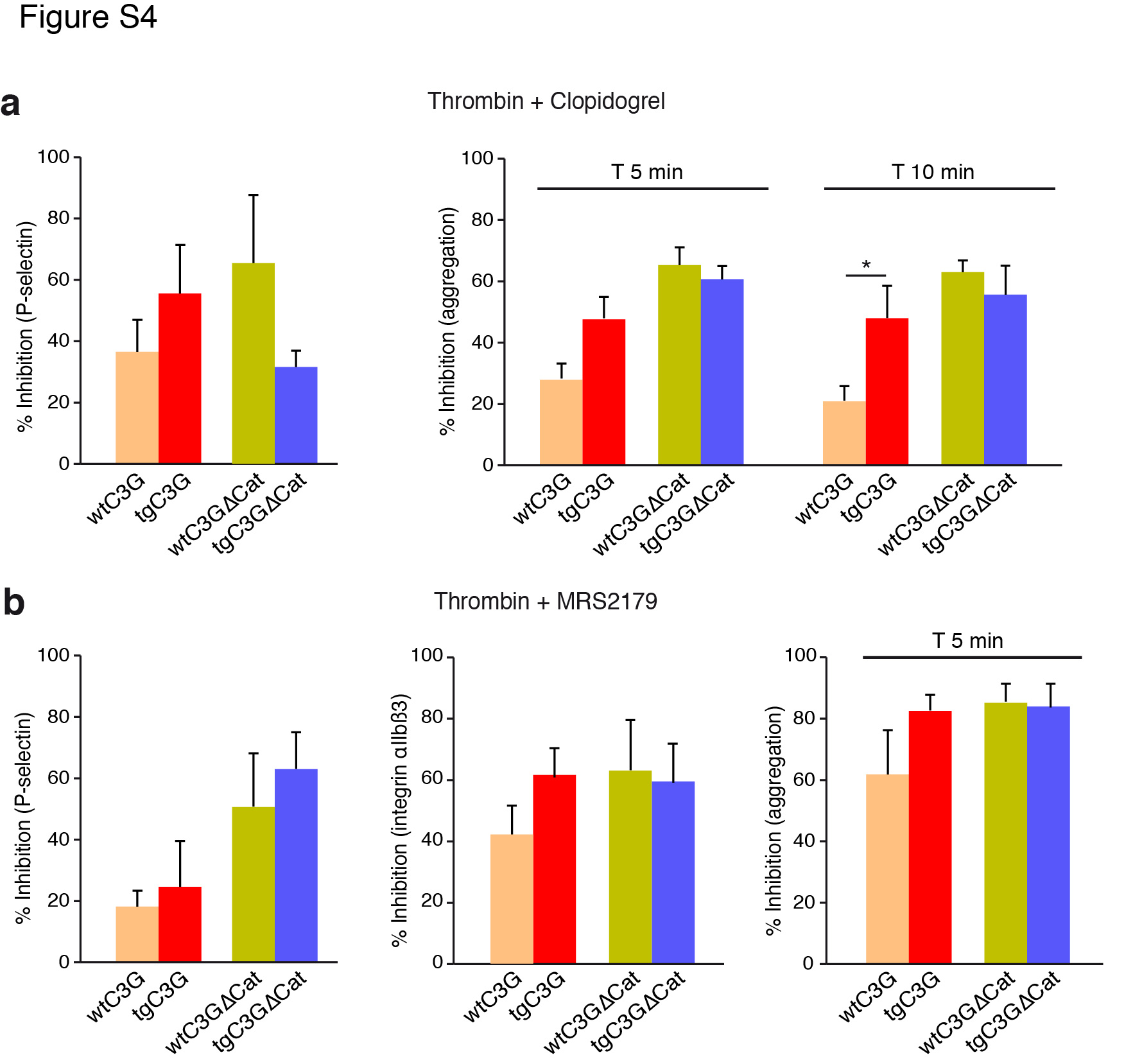


**Figure S4. Analysis of the differential sensitivities of** **tgC3G and tgC3GΔCat platelets to inhibitors of the P2Y12 and P2Y1 receptors**. Platelets from mice of the different genotypes under study were pre-treated with (**a**) 100 μM clopidogrel or (**b**) 100 μM MRS2179 for 5 min and then, stimulated with 1 U/ml thrombin (15 min for activation, 5 min or 10 min for aggregation), as indicated. The histograms represent the mean ± SEM (n>4) of the percentages of inhibition of the expression of P-selectin on the surface, activation of integrin αIIbβ3, or aggregation, in platelets treated with thrombin + inhibitor, compared to thrombin-treated platelets. tg: transgenic; wt: wild type; T: thrombin.

**Figure S5**

**
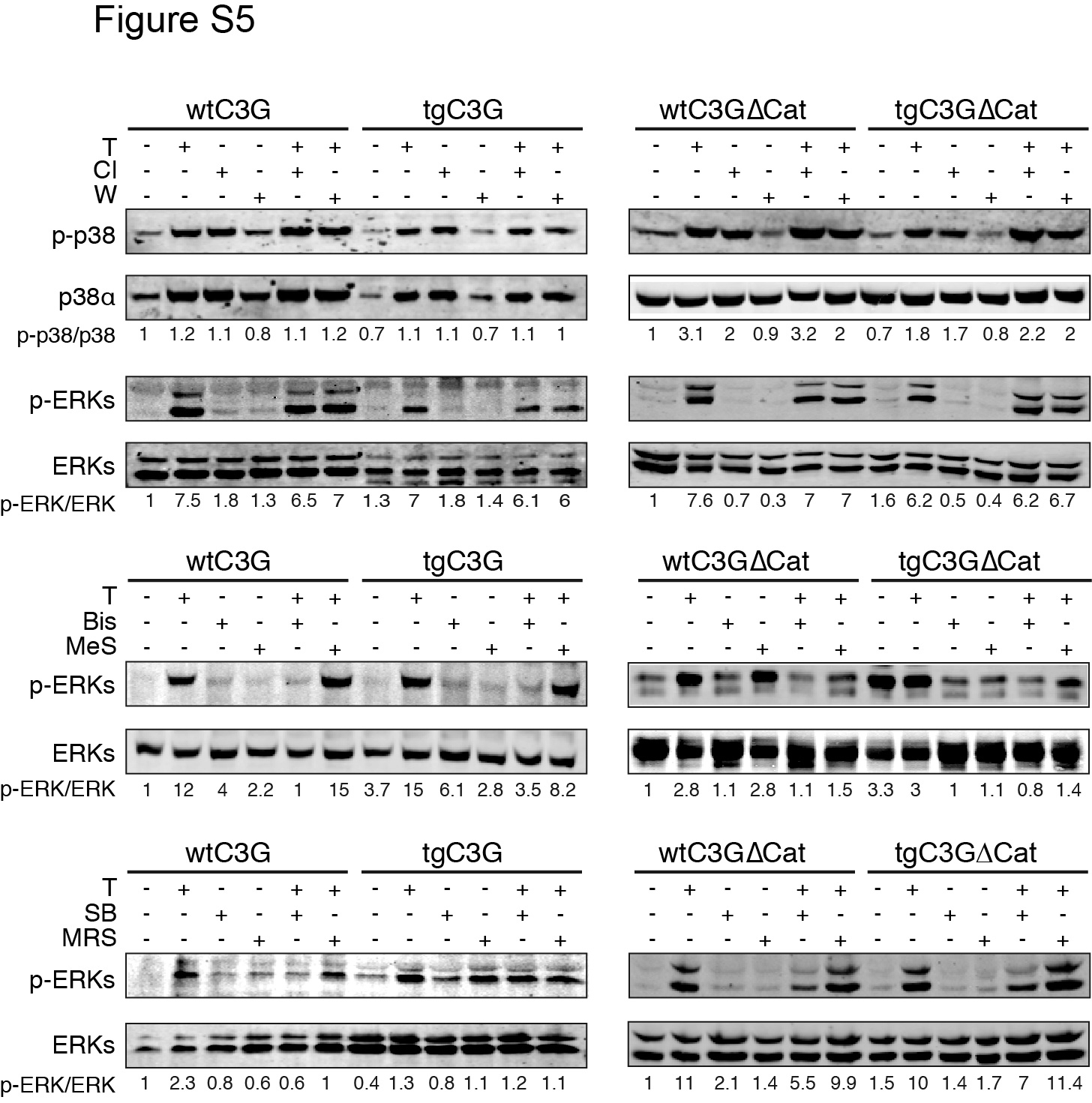
**

**Figure S5. Thrombin-induced ERK and p38 MAPK phosphorylation is independent of C3G.** Platelets from mice of the different genotypes under study were pre-treated with 100 μM clopidogrel (Cl), 100 nM wortmannin (W), 5 μM bisindolylmaleimide (BIS), 50 μM 2-MeSAMP (MeS), 20 μM SB203580 (SB) or 20 μM MRS2179 (MRS) for 5 min and then, stimulated with 1 U/ml thrombin for 1 min at RT. Proteins were detected with antibodies against p-ERKs, ERKs, p-p38 and p38α. Values, indicated beneath the blots, are relative to non-treated platelets and normalized against total ERKs or p38α. Tg: transgenic; wt: wild type; T: thrombin.

**Figure S6**

**
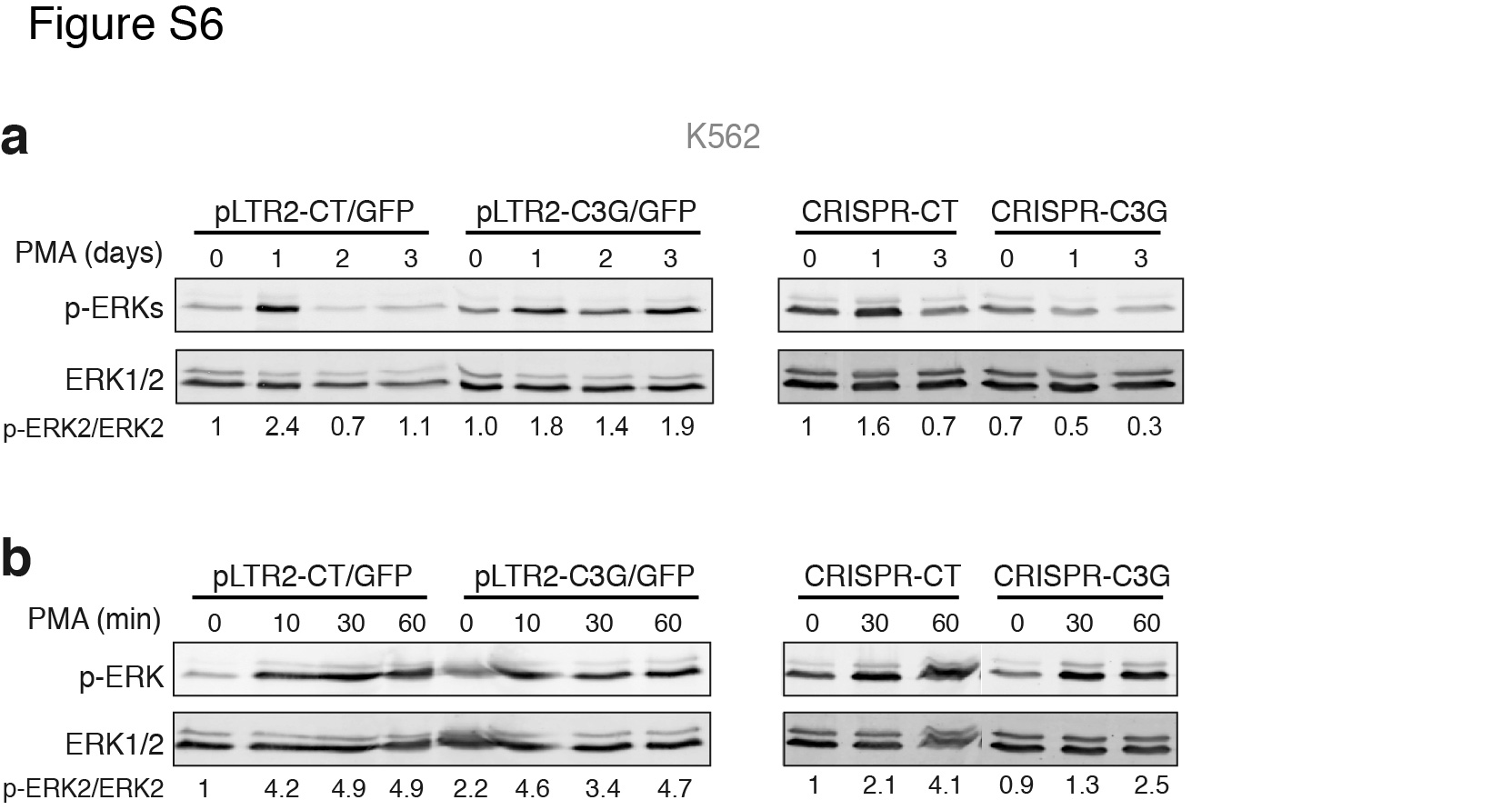
**

**Figure S6. C3G contributes to the sustained activation of ERKs.** Time course of ERK activation by the analysis of the phosphorylated ERK levels (p-ERK) by western-blotting, in C3G-overexpressing (pLTR2-C3G/GFP) and C3G knockout (CRISPR-C3G) K562 clones and their controls (pLTR2-CT/GFP and CRISPR-CT), treated with 20 nM PMA for 1, 2 and 3 days (**a**) or for 10, 30 and 60 min (**b**), using anti phospho-ERKs antibodies. The expression of total ERKs was used as a loading control. Relative p-ERKs/ERKs ratios are shown. All values are relative to control, non-treated cells. All these constructs have been described in (*3*).

**Figure S7**


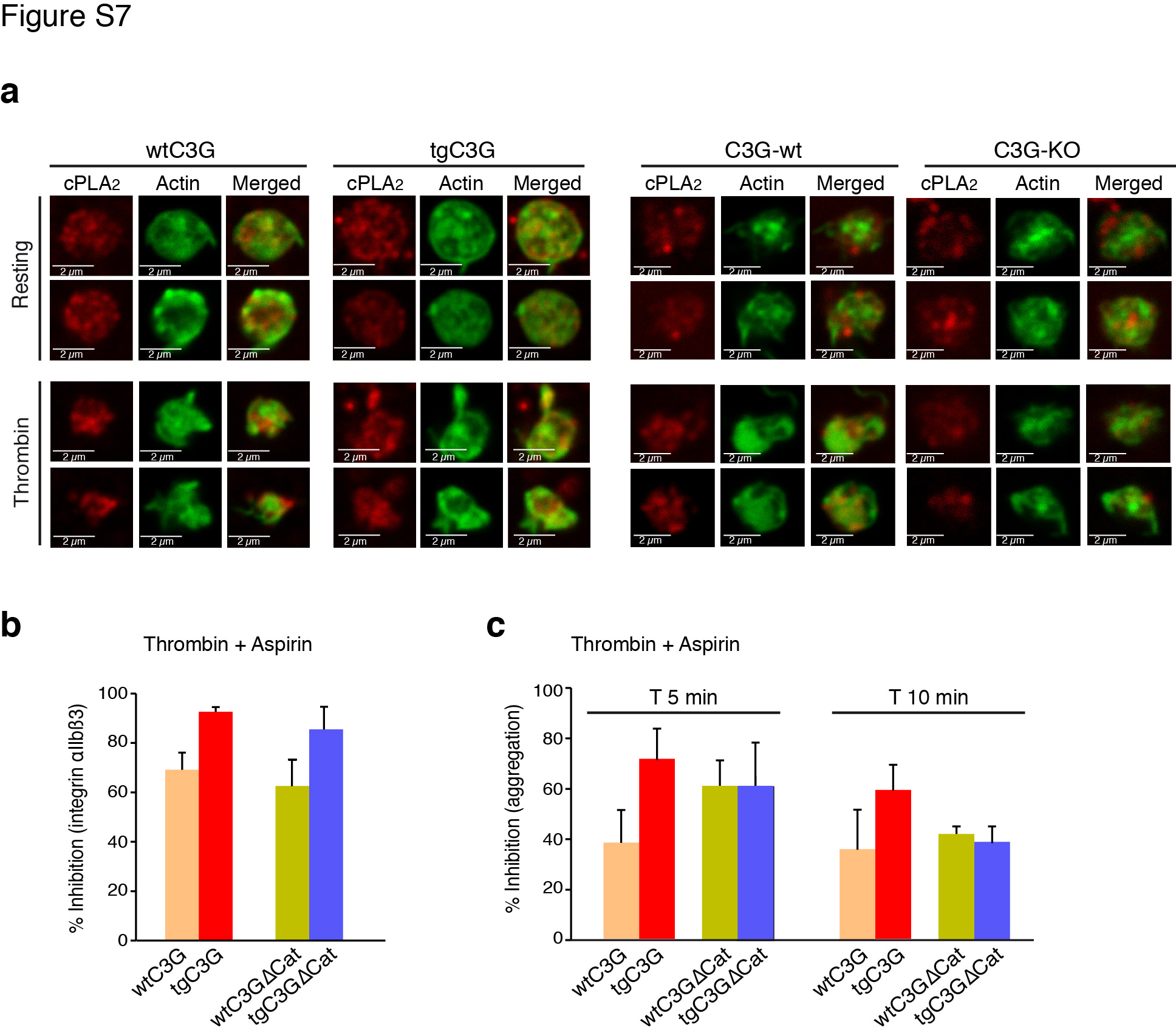


**Figure S7. C3G participates in the TXA_2_ pathway in platelets.** (**a**) Representative immunofluorescence confocal microscopy images of tgC3G and C3G-KO platelets and their corresponding controls, treated with 0.2 U/ml or 1 U/ml thrombin, respectively, and stained with anti-cPLA_2__Alexa Fluor 647 (red) and phalloidin (green). Images of wtC3G/tgC3G and C3G-wt/C3G-KO correspond to two different experiments, but all images from each experiment were taken at the same exposure time. Bar: 2 μm. The histograms represent the mean ± SEM of the percentage of inhibition of the activation of integrin αIIbβ3 (**b**) or aggregation (**c**) in platelets treated with thrombin + aspirin compared to agonist-treated platelets. Aggregation was induced by stimulation with thrombin (T) for 5 or 10 min. 4 to 6 mice of each genotype were used.
